# Differential homotypic and heterotypic interactions of antigen 43 (Ag43) variants in autotransporter-mediated bacterial autoaggregation

**DOI:** 10.1038/s41598-019-47608-4

**Published:** 2019-07-31

**Authors:** Valentin Ageorges, Marion Schiavone, Grégory Jubelin, Nelly Caccia, Philippe Ruiz, Ingrid Chafsey, Xavier Bailly, Etienne Dague, Sabine Leroy, Jason Paxman, Begoña Heras, Frédérique Chaucheyras-Durand, Amanda E. Rossiter, Ian R. Henderson, Mickaël Desvaux

**Affiliations:** 1 Université Clermont Auvergne, INRA, UMR454 MEDiS, 63000 Clermont-Ferrand, France; 2 Lallemand Animal Nutrition, 31702 Blagnac, Cedex France; 3 LISBP, Université de Toulouse, CNRS UMR5504, INRA UMR792, INSA, 31077 Toulouse, Cedex France; 4 LAAS-CNRS, Université de Toulouse, 31077 Toulouse, Cedex France; 5 INRA UR346 Epidémiologie Animale, 63122 Saint Genès Champanelle, France; 6 Department of Biochemistry and Genetics, La Trobe Institute for Molecular Science, La Trobe University, Bundoora, Victoria 3086 Australia; 7 Institute of Microbiology and Infection, University of Birmingham, B152TT Birmingham, United Kingdom; 8 Institute for Molecular Biosciences, University of Queensland, St. Lucia, Queensland 4067 Australia

**Keywords:** Molecular biology, Microbiology, Bacterial secretion

## Abstract

Antigen 43 (Ag43) is a cell-surface exposed protein of *Escherichia coli* secreted by the Type V, subtype a, secretion system (T5aSS) and belonging to the family of self-associating autotransporters (SAATs). These modular proteins, comprising a cleavable N-terminal signal peptide, a surface-exposed central passenger and an outer membrane C-terminal translocator, self-recognise in a Velcro-like handshake mechanism. A phylogenetic network analysis focusing on the passenger revealed for the first time that they actually distribute into four distinct classes, namely C1, C2, C3 and C4. Structural alignment and modelling analyses demonstrated these classes arose from shuffling of two different subdomains within the Ag43 passengers. Functional analyses revealed that homotypic interactions occur for all Ag43 classes but significant differences in the sedimentation kinetics and aggregation state were present when Ag43^C3^ was expressed. In contrast, heterotypic interaction occurred in a very limited number of cases. Single cell-force spectroscopy demonstrated the importance of specific as well as nonspecific interactions in mediating Ag43-Ag43 recognition. We propose that structural differences in the subdomains of the Ag43 classes account for different autoaggregation dynamics and propensities to co-interact.

## Introduction

In lipopolysaccharidic (LPS) diderm bacteria (archetypical Gram-negative bacteria), nine protein secretion systems (T1SS-T9SS) are currently recognised as enabling protein transport across the outer membrane (OM), whereas the Sec and Tat export pathways enable protein translocation across the cytoplasmic membrane, also called the inner membrane (IM)^1–3^. Among those, the T5SS represents the simplest and most widely used mechanism for protein secretion in LPS-diderm bacteria^4–7^, where proteins are translocated across the OM via a β-barrel with the assistance of some periplasmic chaperones (e.g. Skp, SurA or DegP), the BAM (β-barrel assembly machinery) and TAM (translocation and assembly module) complexes along the process^8–11^. Based on phylogenetic analyses and structural features, the T5SS is subdivided into the (i) classical autotransporters (T5aSS), (ii) two-partner passenger-translocators (T5bSS), (iii) trimeric autotransporters (T5cSS), (iv) hybrid autotransporters (T5dSS), and (v) inverted autotransporters (T5eSS)^11–14^.

A classical autotransporter (AT) is a modular protein constituted of three main regions, i.e. (i) a N-terminal signal peptide (SP), (ii) a central passenger, and (iii) a translocator at the C-terminus^15,16^. As such, AT export via the Sec translocase is mediated by the SP before the passenger is secreted through the β-barrel pore formed by the translocator in the OM. Once secreted, the passenger can have three different fates as it may be (i) displayed at the bacterial cell surface, (ii) cleaved from the translocator and released as an exoprotein into the extracellular milieu, or (iii) processed but remain associated to the cognate translocator^10^. The functions of ATs are quite diverse and can be broadly categorised as (i) self-associating ATs (SAATs), (ii) protease ATs, (iii) phosphatase/hydrolase ATs, (iv) lipase/esterase ATs, (v) vacuolating ATs, (vi) adhesin ATs, and (vii) ATs of unknown function^3,10^. SAATs promote bacterial aggregation by cell self-association, a phenomenon called autoaggregation^17,18^. Besides flocculation and sedimentation of bacterial cells, autoaggregation is an important mechanism with consequences for bacterial virulence and infection due to biofilm formation, protection from environmental stresses, antibiotic resistance and defence against predators^18^. The antigen 43 (Ag43) is a prototypical member of the SAATs; this category of ATs also include AIDA (adhesin involved in diffuse adherence) and TibA (toxigenic invasion locus b)^17^. Besides homotypic interactions, heterotypic interactions were demonstrated between AIDA-Ag43, AIDA-TibA and Ag43-TibA^17,19^. By promoting interspecies cell-to-cell contact, these interactions would have important ecophysiological roles in inducing co-operative multispecies biofilms that allows for more successful microbial colonisation of various niches^20^.

Besides the basic and common modular architecture of an AT, Ag43 exhibits some additional features. At the N-terminus, Ag43 displays a highly conserved extended SP region (ESPR) that slows the rate of export across the IM through the Sec-YidC pathway to prevent protein misfolding in the periplasm and consequently sustain the secretion-competent state of the passenger across the OM through the translocator^21,22^. Of note, the ESPR is not exclusive to Ag43 as it occurs in many different, but not all, ATs. N-terminal to the translocator, Ag43 exhibits a type 1 autochaperone domain (AC1), which is considered as essential for folding of β-helical passengers^3,23^. Like most ATs, the Ag43 passenger folds into a single-stranded right-handed parallel β-helix with triangular-shaped coil cross-sections^3,24^. Most recently, the crystal structure of the Ag43 from uropathogenic *E*. *coli* (UPEC) CFT073 (Ag43a) was solved and revealed the passenger displayed a twisted L-shape β-helical architecture^25^. The L-shaped conformation of the passenger further appeared as a requirement for driving the formation of cell aggregates and self-association in a Velcro-like handshake mechanism. While the function of Ag43 was primarily investigated in *Escherichia coli* K12^24^, its implication in autoaggregation, adhesion and biofilm formation was further confirmed in some pathogenic species, namely enterohaemorrhagic *E*. *coli* (EHEC) and UPEC^26,27^. The Ag43 is encoded by two identical alleles in EHEC O157:H7 EDL933 but by two different alleles in UPEC CFT073; the UPEC Ag43a variant mediates a strong autoaggregation and biofilm formation phenotypes, whereas the Ag43b variant is a less efficient aggregating and biofilm forming factor^27^. Phylogenetic analyses had revealed the existence of two subfamilies (SF) of Ag43, namely SF-I and SF-II^24^.

While homotypic Ag43 interactions have previously been noted, heterotypic interactions of Ag43 variants had not been extensively characterised as yet, it prompted us to investigate these aspects in a systematic study. In a cooperative evolution hypothesis that “birds of a feather flock together”, different combinations of Ag43 variants might select bacterial interactions with siblings to the exclusion of others. From phylogenetic analyses of Ag43 passengers, it first appeared that the grouping was not dichotomous but clustered into four different classes associated to structural differences. Following the expression of representative Ag43 members, different propensities of the variants to induce autoaggregation were evidenced. By monitoring the formation of cell aggregates by epifluorescence microscopy and flow cytometry, homotypic and heterotypic interactions between different Ag43 variants were for the first time identified and characterised. Moreover, biophysical studies by atomic force microscopy (AFM) revealed different dynamics of Ag43 interactions suggesting that structural differences in the N-terminal and C-terminal subdomains of the Ag43 passengers can modulate the association of the different variants.

## Results

### Ag43 passengers cluster into four distinct phylogenetic classes with combination of two different subdomains

**Figure 1:**
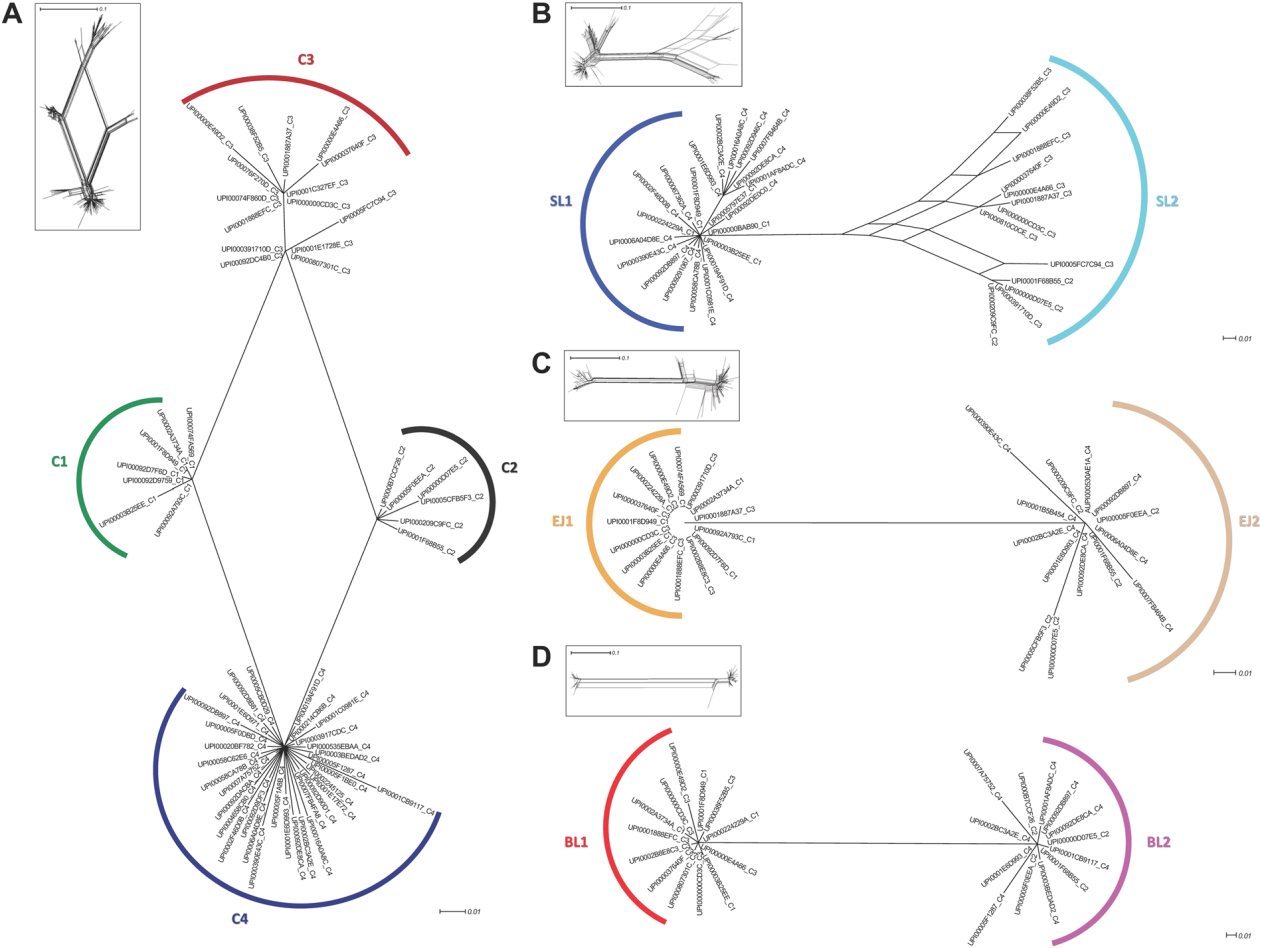
Phylogenetic analysis of Ag43 passengers and their subdomains. The amino acid sequences of the passengers were aligned using T-Coffee and structural information of Ag43^C3-UPI00000E4A66^ (PDB: 4KH3) as template. Based on the L-shaped Ag43 structure (Fig. 2), the amino acid sequences of the SL, EJ and BL subdomains were recovered for alignment. Trees were generated using SplitsTree applying bootstrapping and 95% confidence for the observed nodes. Inserts represent the trees before applying bootstrapping and confidence threshold as provided in Supplementary Fig. S1. (**A**) Tree of the Ag43 passengers highlighting the four classes, C1, C2, C3 and C4. (**B**) Tree of the SL subdomain highlighting the two subtypes, SL1 and SL2. (**C**) Tree of the EJ subdomain highlighting the two subtypes, EJ1 and EJ2. (**D**) Tree of the BL subdomain highlighting the two subtypes, BL1 and BL2. For clarity, some sequences were removed from the trees but are readily available in Supplementary Table S1 and Fig. S1. The scale bars represent the number of substitutions per site.

The functional properties of the modular protein Ag43 are determined by the passenger, which protrudes from the bacterial OM upon translocation. To apprehend the diversity of Ag43, a phylogenetic network analysis of the passenger was applied to a dataset of 106 distinct passengers generated from previously reported Ag43 sequences in the literature^24,28^ (Supplementary Table S1). The network obtained defined for four main classes of passengers (C1 to C4) supported by robust splits (Fig. 1A).

C1 harbours the Ag43 passenger from *E*. *coli* K12 MG1655 (UPI00003B25EE), which is among the first identified and the most investigated Ag43^24^. C2 includes the Ag43 passenger from EHEC O157:H7 EDL933 (UPI00000D07E5), which is the first Ag43 investigated in a pathogenic *E*. *coli*^26^. C3 harbours the Ag43a (UPI00000E4A66) and Ag43b (UPI00000E49D2) from UPEC CFT073, the former being the first and only structurally characterised Ag43 passenger (PDB: 4KH3)^25^. To date, no Ag43 homologues from C4 have been functionally or structurally characterised. The original SF-I grouping corresponds to the gathering of C1 and C3, whereas SF-II grouping reflects C2 and C4^24^. The initial clustering correlated with the most obvious difference among the Ag43 passengers, their sizes; while Ag43 passengers from C1 and C3 contain around 500 amino acids, C2 and C4 homologues are smaller with about 430 amino acids (Supplementary Table S1).

**Figure 2:**
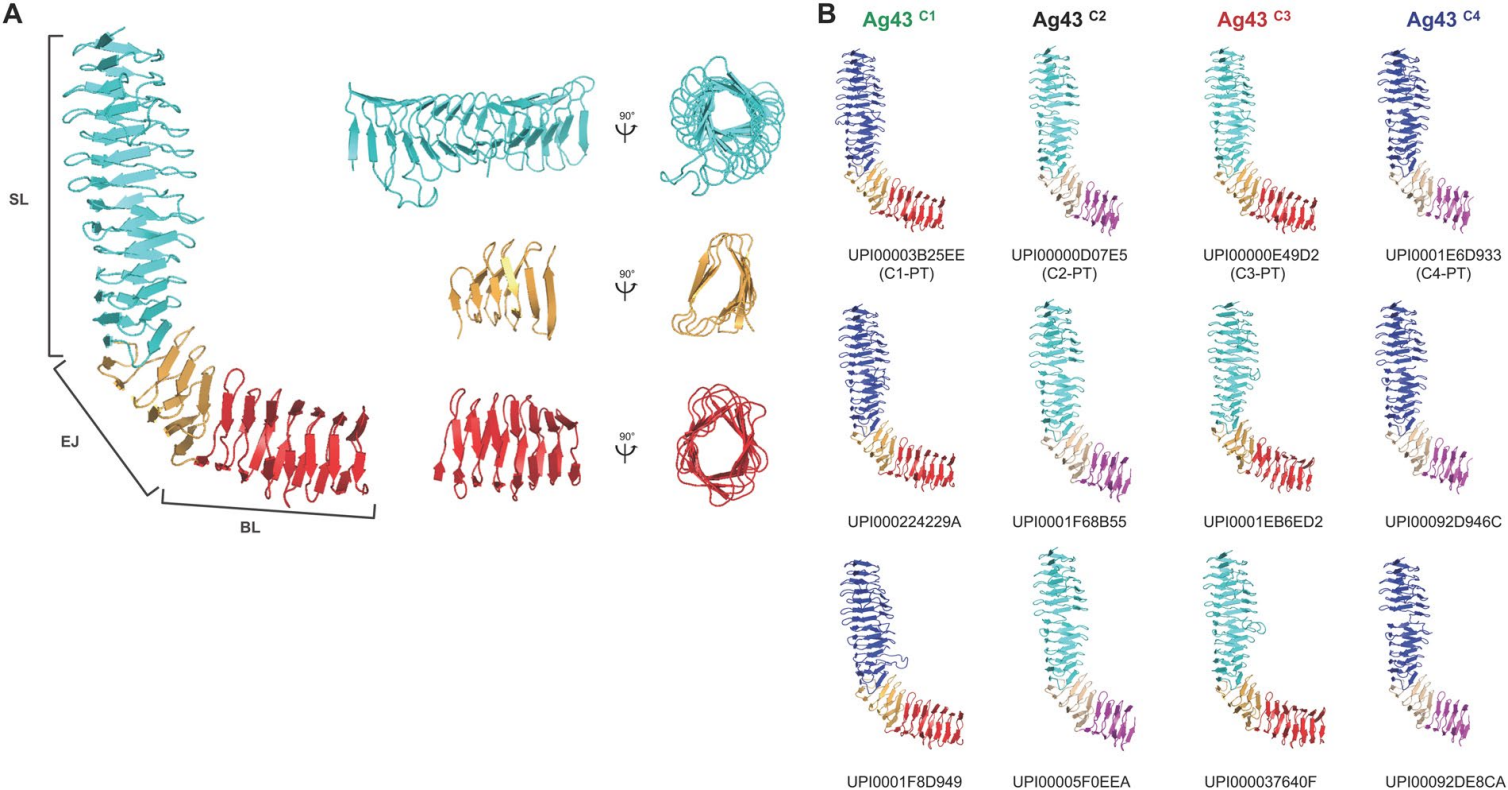
Modular structure of the Ag43 passengers belonging to classes 1, 2, 3 and 4. (**A**) Based on the experimentally determined L-shaped structure of Ag43^C3-UPI00000E4A66^ (PDB: 4KH3), three subdomains could be defined SL (for stem of the letter L), EJ (for elbow joint), and BL (for bottom of the letter L)^25^. Both SL and BL subdomains exhibit a right-handed parallel β-helix, the cross-sections of each rung along SL form triangular (T) β-solenoids of subtype 4 (T4) and of T5-type β-solenoids for BL (Supplementary Table S1). The EJ subdomain is flanked by β-hairpin motifs with three-rung parallel T5 β-solenoids. (**B**) Molecular modelling of the structures of Ag43 passengers from C1 to C4 using Phyre^54,55^. The SL domain is shown in shades of blue, with electric blue corresponding to SL1 and cyan to SL2. The EJ domain is depicted in shades of orange, with bright orange corresponding to EJ1 and light orange to EJ2. The BL domain is represented in shades of red, with bright red corresponding to BL1 and magenta to BL2. PT: prototypical variant.

Our updated phylogenetic network further revealed a strong ambiguity in the relatedness patterns among Ag43 passengers from C1, C2, C3, and C4, illustrated by the diamond shape of the network with almost no specific divergence. Therefore, a more detailed analysis of subdomains of the passengers was performed to identify the source of this ambiguity. Homologies to pectin lyase fold (IPR011050) overlapping with P22 tailspike protein (IPR012332) and AT adhesin (IPR030930) domains were systematically predicted for all Ag43 passengers, highlighting the β-helix topology encountered in SAATs (Supplementary Table S1). Modelling of the 3D structure of the Ag43 passengers confirmed that they are all predicted to exhibit a right-handed parallel β-helix fold with an L-shaped structure as originally observed in Ag43^C3-UPI00000E4A6625^ (Fig. 2A). Following the analysis of the coil cross-sections along the Ag43 passengers^29^, it appeared the N-terminal part, namely the SL (for stem of the letter L) region, systematically exhibited triangular (T) β-solenoids of subtype 4 (T4) (Fig. 2 and Supplementary Table S1). A phylogenetic network analysis of the SL subdomain identified two robust and unambiguously differentiated clusters of sequences, SL of type 1 (SL1) and SL2 (Fig. 1B). The two subtypes were related by a single branch, and remaining splits were located within SL2. Sequences from SL1 and SL2 subdomains had mean percentages of similarity of 93 and 88%, respectively, but these percentages fell at 75% when comparing SL1 versus SL2 (Supplementary Table S1). While SL1 clustered subdomains corresponding to Ag43^C1^ and Ag43^C4^ passengers, SL2 clustered subdomains from Ag43^C2^ and Ag43^C3^ passengers. Nevertheless, the SL region appeared slightly longer by 15–17 amino acids in Ag43^C1^ and Ag43^C3^ passengers.

The elbow region joining (EJ) the upper and lower parts of the L-shape was always bordered by β-hairpin motifs with three-rung parallel T5 β-solenoids in-between, which exhibited a highly conserved sequence in the first rung, namely VVLE[N/S]GGRLDVL. The EJ region reveals a remarkably conserved size of 85 amino acids. The phylogenetic network analysis of the EJ subdomain showed a different relatedness pattern compared to the SL subdomain. Two robust sequence subtypes were clustered, namely (i) EJ of type 1 (EJ1), which included Ag43 passenger subdomains from C1 and C3, and (ii) EJ2, which clustered subdomains from the C2 and C4 (Fig. 1C). In average, sequences from EJ1 and EJ2 subdomains had percentages of similarity of 97 and 94%, respectively, but these percentages fell at 53% when comparing EJ1 versus EJ2 (Supplementary Table S1).

The BL (for bottom of the letter L) subdomain in the C-terminal part of the passengers is composed of T5-type β-solenoids (Fig. 2 and Supplementary Table S1). The phylogenetic network analysis of the BL was coherent with the analysis of the EJ subdomain. Two robust sequence subtypes were clustered: (i) BL of type 1 (BL1), which included Ag43 passengers subdomains from C1 and C3, and (ii) BL2, which clustered subdomains from C2 and C4 (Fig. 1D). Sequences from BL1 and BL2 subdomains had percentages of similarity from and above 95 and 93% (mean values of 99 and 98%), respectively, but these percentages fell below 30% (28% in average) when comparing SL1 versus SL2 (Supplementary Table S1). Furthermore, a difference of 53 amino-acids among the length of BL subdomains was observed. It accounted for most of the observed variation in the length of Ag43 passengers between C1/C3 over C2/C4 (Supplementary Table S1). This difference indeed resulted from a truncation at the C-terminus of BL2 compared to BL1.

These data suggest that the two SL phylogenetic clusters and the two coherent phylogenetic clusters of EJ and BL were shuffled during the evolution of Ag43 proteins explaining the observed ambiguity in relatedness pattern of the passengers (Fig. 1A). It resulted in four combinations of Ag43 passengers subdomains: (i) SL1-EJ1-BL1 corresponding to Ag43^C1^, (ii) SL2-EJ2-BL2 corresponding to Ag43^C2^, (iii) SL2-EJ1-BL1 corresponding to Ag43^C3^, and (iv) SL1-EJ2-BL2 corresponding to Ag43^C4^.

### Autoaggregation occurs through homotypic interactions for all Ag43 variants but the size of the aggregates is significantly much smaller with Ag43^C3^

**Figure 3:**
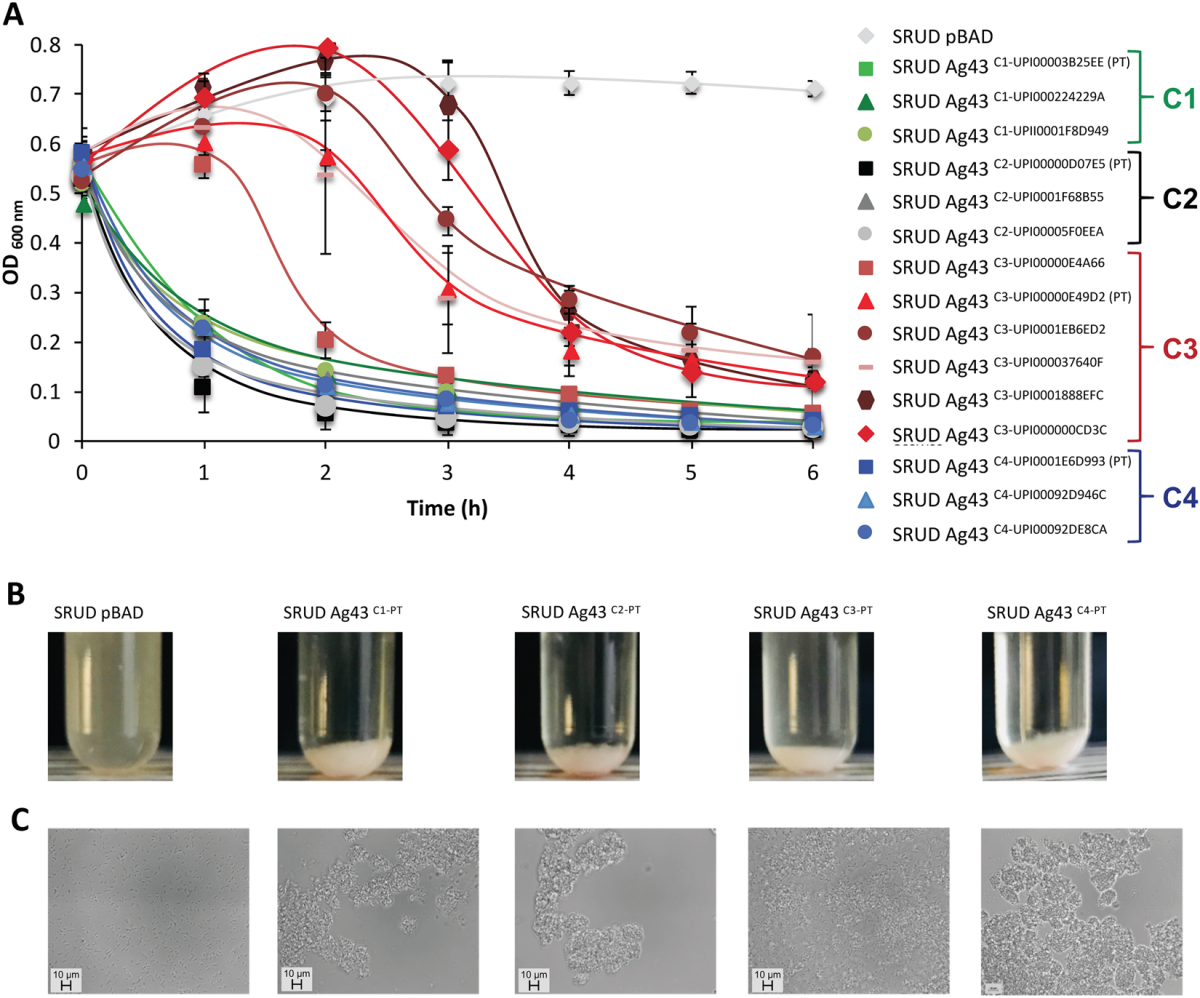
Autoaggregation of *E*. *coli* SRUD expressing different Ag43 variants. (**A**) The sedimentation assay was performed in LB at 37 °C over 6 h with SRUD expressing variants of Ag43 from C1 (shades of green), Ag43^C2^ (shades of grey), (iii) Ag43^C3^ (shades of red), Ag43^C4^ (shades of blue). The nonaggregative SRUD pBAD was used as negative control. (**B**) Sedimentation was visualised after 6 h of static incubation. (**C**) Phase-contrast microscopy observation from sampling at 6 h of static incubation. PT: prototypical variant (Table 1).

Our new classification of Ag43 delineated the proteins into four classes based on sequence similarity and consequently structural differences. We hypothetised that these variations would result in functional differences. Therefore, we investigated the autoaggregation phenotype of three selected Ag43 homologues from each of the four classes (Fig. 2 and Table 1) when expressed in the non-aggregative *E*. *coli* SRUD^30^. When bacterial cell cultures were left standing statically, all bacterial cells expressing the Ag43 flocculated and sedimented at the bottom of tubes contrary to the control SRUD pBAD (with empty vector pBAB/Myc-HisA, i.e. Ag43^−^) (Fig. 3A,B) (Table 1). No significant differences could be observed for the sedimentation kinetics of bacterial cells expressing Ag43 from C1, C2 and C4 with all three having compact sedimentation pellets. Microscopic observations indicated the flocculation and sedimentation of the bacterial cultures resulted from bacterial cell autoaggregation as no cell aggregates could be observed with SRUD pBAD, control (Fig. 3C).

**Table 1:** Genotype and phenotype of most relevant *E*. *coli* strains used in the study.

Name of *E*. *coli* strain^a^	Relevant genotype and/or phenotype
SRUD	*E*. *coli* K12 MG1655 *∆fim ∆agn43*^30^
SRUD-Green pBAD	Empty pBAD/Myc-HisA in *E*. *coli* SRUD-Green
SRUD-Red pBAD	Empty pBAD/Myc-HisA in *E*. *coli* SRUD-Red
SRUD-Blue pBAD	Empty pBAD/Myc-HisA in *E*. *coli* SRUD-Blue
SRUD Ag43^C1-PT^	pBAD-Ag43^C1-UPI00003B25EE^ in *E*. *coli* SRUD
SRUD-Green Ag43^C1-PT^	pBAD-Ag43^C1-UPI00003B25EE^ in *E*. *coli* SRUD-Green
SRUD-Red Ag43^C1-PT^	pBAD-Ag43^C1-UPI00003B25EE^ in *E*. *coli* SRUD-Red
SRUD-Blue Ag43^C1-PT^	pBAD-Ag43^C1-UPI00003B25EE^ in *E*. *coli* SRUD-Blue
SRUD Ag43^C2-PT^	pBAD-Ag43^C2-UPI00000D07E5^ in *E*. *coli* SRUD
SRUD-Green Ag43^C2-PT^	pBAD-Ag43^C2-UPI00000D07E5^ in *E*. *coli* SRUD-Green
SRUD-Red Ag43^C2-PT^	pBAD-Ag43^C2-UPI00000D07E5^ in *E*. *coli* SRUD-Red
SRUD-Blue Ag43^C2-PT^	pBAD-Ag43^C2-UPI00000D07E5^ in *E*. *coli* SRUD-Blue
SRUD Ag43^C3-PT^	pBAD-Ag43^C3-UPI00000E49D2^ in *E*. *coli* SRUD
SRUD-Green Ag43^C3-PT^	pBAD-Ag43^C3-UPI00000E49D2^ in *E*. *coli* SRUD-Green
SRUD-Red Ag43^C3-PT^	pBAD-Ag43^C3-UPI00000E49D2^ in *E*. *coli* SRUD-Red
SRUD-Blue Ag43^C3-PT^	pBAD-Ag43^C3-UPI00000E49D2^ in *E*. *coli* SRUD-Blue
SRUD Ag43^C4-PT^	pBAD-Ag43^C4-UPI0001E6D993^ in *E*. *coli* SRUD
SRUD-Green Ag43^C4-PT^	pBAD-Ag43^C4-UPI0001E6D993^ in *E*. *coli* SRUD-Green
SRUD-Red Ag43^C4-PT^	pBAD-Ag43^C4-UPI0001E6D993^ in *E*. *coli* SRUD-Red
SRUD-Blue Ag43^C4-PT^	pBAD-Ag43^C4-UPI0001E6D993^ in *E*. *coli* SRUD-Blue

^a^The complete list of *E*. *coli* strains and plasmids used in this study is available in Supplementary Table S2. PT: prototypical variant.

In contrast, strains expressing the three C3 homologues (Fig. 2), namely Ag43^C3-UPI00000E4A66^, Ag43^C3-UPI00000E49D2^ and Ag43^C3-UPI0001EB6ED2^, showed a significant delay in the autoaggregation kinetics compared to other Ag3 expressing strains (Fig. 3A). In addition, the sedimentation pellets were looser and cell aggregates appeared smaller, with the presence of individual single cells (Fig. 3B,C). Within C3, some significant differences were observed as bacterial cultures of SRUD expressing Ag43^C3-UPI00000E4A66^ sedimented significantly quicker than those expressing Ag43^C3-UPI00000E49D2^ or Ag43^C3-UPI0001EB6ED2^. To confirm this slower autoaggregation phenotype for bacterial cells expressing Ag43^C3^, three additional homologues from this class were tested; in each case, autoaggregation was slower (Fig. 3A).

**Figure 4:**
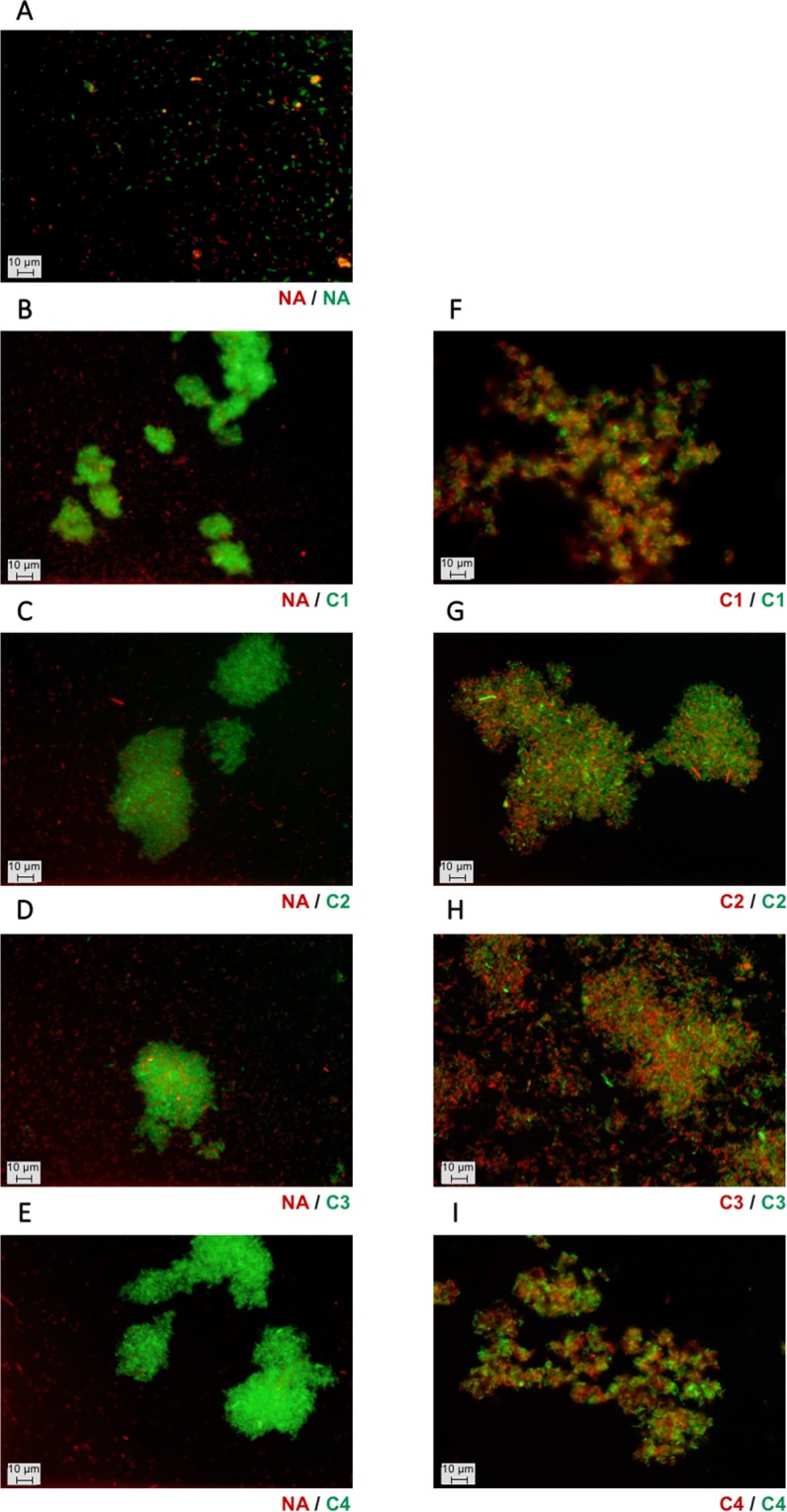
Fluorescent microscopy analysis of homotypic interactions of bacterial cells expressing different variants of Ag43. Epifluorescence microscopic observations were performed from bacterial cells grown in LB and after 6 h of static incubation at 37 °C. Co-culture of nonaggregative (NA) SRUD-Red pBAD with (**A**) SRUD-Green pBAD, (**B**) SRUD-Green Ag43^C1-PT^, (**C**) SRUD-Green Ag43^C2-PT^, (**D**) SRUD-Green Ag43^C3-PT^, or (**E**) SRUD-Green Ag43^C4-PT^. Co-culture of SRUD-Red and SRUD-Green both expressing (**F**) Ag43^C1-PT^, (**G**) Ag43^C2-PT^, (**H**) Ag43^C3-PT^, or (**I**) Ag43^C4-PT^. The NA, C1, C2, C3 and C4 labels match the colour of the fluorescent bacterial strains.

To further characterise the homotypic interactions that are responsible for the autoaggreagtion phenotype, one prototypical (PT) representative was selected from each class (Table 1), namely Ag43^C1-PT^ (UPI00003B25EE), Ag43^C2-PT^ (UPI00000D07E5), Ag43^C3-PT^ (UPI00000E49D2) and Ag43^C4-PT^ (UPI0001E6D993). Fluorescence microscopy revealed that in the absence of Ag43 (Ag43^−^, i.e. SRUD-Green pBAD and SRUD-Red pBAD) no cell aggregates could be observed (Fig. 4A). In contrast aggregates were detected for cells expressing Ag43 variants from each group (Fig. 4B–E). The combination of Ag43^−^ red-fluorescent-tagged cells mixed with Ag43^+^ green-fluorescent-tagged cells (i.e. SRUD-Green pBAD-Ag43) systematically exhibited only one type of bacterial cell aggregate, consisting uniquely of green-fluorescent-tagged cells where only a few Ag43^−^ cells could be observed as trapped within the aggregates (Fig. 4B–E). When green- and red-fluorescent cells expressing the same Ag43 were mixed equally, both fluorescent cells were found in the aggregates (Fig. 4F–I). These results confirmed that all tested Ag43 proteins, Ag43^C1-PT^, Ag43^C2-PT^, Ag43^C3-PT^ or Ag43^C4-PT^, enable homotypic interactions inducing bacterial autoaggregation. However, examination of the obtained micrographs seemed to indicate that more individual cells were present when Ag43^C3-PT^ was expressed (Fig. 4H).

**Figure 5:**
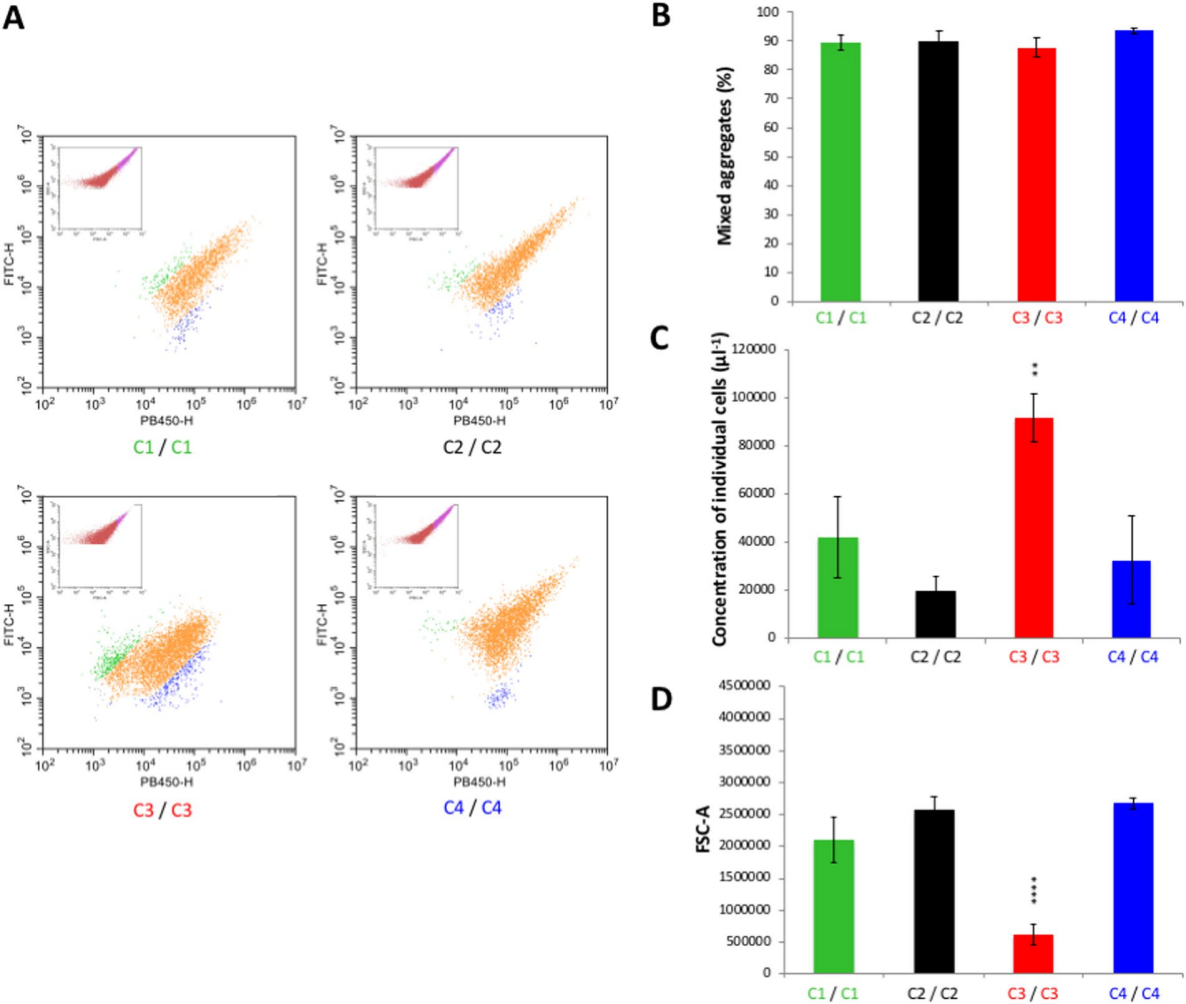
Characterisation of homotypic interactions of Ag43 variants by flow cytometry. Flow cytometry analyses were performed by mixing SRUD-Green and SRUD-Blue both expressing the same Ag43, namely Ag43 ^C1-PT^ (C1), Ag43^C2-PT^ (C2), Ag43^C3-PT^ (C3) or Ag43^C4-PT^ (C4). (**A**) Based on the SSC-A over FSC-A representation (pictured in the insert), the fluorescent FITC-H over PB450-H representation focused on the gate corresponding to the cell aggregates as previously defined (Supplementary Fig. S3). The green cloud corresponds to cell aggregates of SRUD-Green, the blue cloud to cell aggregates of SRUD-Blue, and the orange cloud to mixed cell aggregates of SRUD-Green and SRUD-Blue. Graphs correspond to one representative experiment. (**B**) The percentage of mixed cell aggregates was calculated from the events detected in orange cloud over the total number of events corresponding to aggregates. (**C**) The concentration of cells was calculated from the gates corresponding to single individual cells as previously defined (Supplementary Fig. S3). (**D**) The size of aggregates was recovered from the gate corresponding to cell aggregates as previously defined (Supplementary Fig. S3).

To gain quantitative insight, fluorescent nonaggregative and aggregative *E*. *coli* cells were analysed using flow cytometry. SSC-A (side scatter area) and FSC-A (forward scatter area) parameters are commonly used to discriminate different types of cells within a heterogeneous population with respect to granularity and cell size, respectively. Here, SSC-A over FSC-A dot plots allowed differentiating single cells from aggregated cells. As shown in Supplementary Fig. S3, flow cytometry analysis of the non-aggregative *E*. *coli* SRUD pBAD revealed a FCS-A/SSC-A pattern that is clearly distinct from the one obtained using the aggregative *E*. *coli* SRUD expressing Ag43^C1-PT^. Comparison of these 2 samples allowed discriminating single cells from cell aggregates, the latter forming a comet tail in the upper part of the SSC-A over FSC-A representation (Supplementary Fig. S3). In agreement with microscopic examination, no cell aggregates were observed in the absence of Ag43, whereas cells expressing Ag43^C1-PT^, Ag43^C2-PT^, Ag43^C3-PT^ and Ag43^C4-PT^ formed aggregates (Supplementary Fig. S4). Furthermore, when green- with blue-fluorescent-tagged SRUD cells expressing the same Ag43 allele were combined, mixed aggregates formed (Fig. 5A); these represented around 90% of the total aggregates (Fig. 5B). As determined by flow cytometry analyses, quantification of the concentration of single individual cells showed a significant difference for cells expressing Ag43^C3-PT^ compared to other Ag43 variants (Fig. 5C). Further, the shorter comet tail for *E*. *coli* SRUD Ag43^C3-PT^ was indicative of smaller aggregates (Fig. 5A) and the mean size of the aggregates was confirmed to be significantly smaller for cells expressing Ag43^C3-PT^ compared to any of the other Ag43 variants (Fig. 5D). To ensure the differences reported for Ag43^C3-PT^ were not influenced by the protein expression levels, Western blot analyses were performed and indicated the Ag43 variants were expressed at similar levels in *E*. *coli* SRUD and displayed at the OM (Fig. S5).

### Autoaggregation occurs only for some combinations of heterotypic interactions

**Figure 6:**
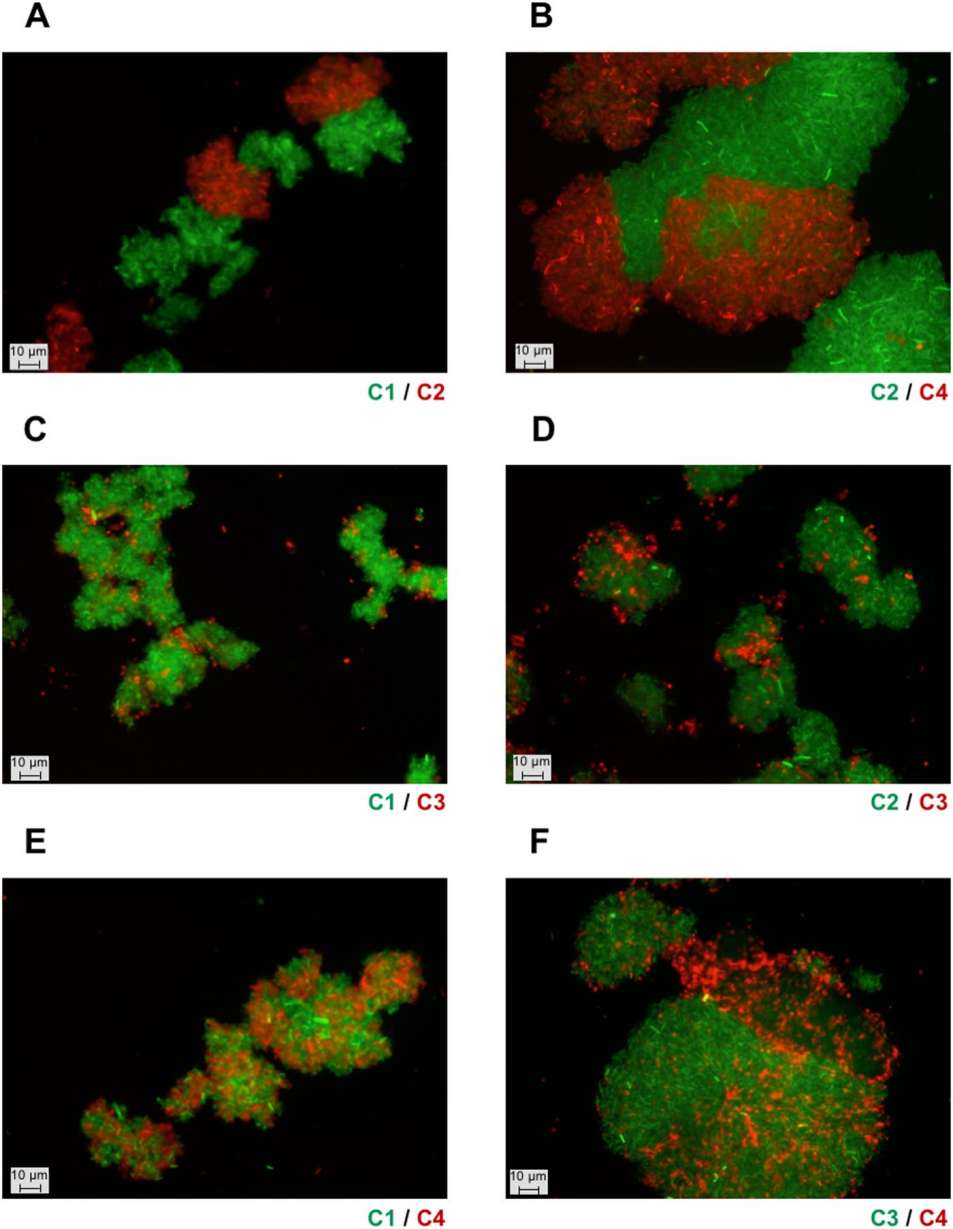
Fluorescent microscopy analysis of heterotypic interactions of *E*. *coli* cells expressing different variants of Ag43. (**A**) Co-culture of SRUD-Green Ag43^C1-PT^ (C1) and SRUD-Red Ag43^C2-PT^ (C2). (**B**) Co-culture of SRUD-Green Ag43^C2-PT^ and SRUD-Red Ag43^C4-PT^ (C4). (**C**) Co-culture of SRUD-Green Ag43^C1-PT^ and SRUD-Red Ag43^C3-PT^ (C3). (**D**) Co-culture of SRUD-Green Ag43^C2-PT^ and SRUD-Red Ag43^C3-PT^. (**E**) Co-culture of SRUD-Green Ag43^C1-PT^ and SRUD-Red Ag43^C4-PT^. (**F**) Co-culture of SRUD-Green Ag43^C3-PT^ and SRUD-Red Ag43^C4-PT^. The NA, C1, C2, C3 and C4 labels match the colour of the fluorescent bacterial strains.

To study heterotypic interactions, all six possible combinations of interactions between the prototypical Ag43 proteins from C1 to C4 were tested (Fig. 6). Fluorescent microscopic examinations revealed three major trends (i) large clumps of bacteria containing only red- or green-fluorescent-tagged cells, suggesting only homotypic interactions occur (Fig. 6A,B), (ii) large aggregates containing only one type of fluorescent-tagged cell with smaller aggregates of the other tagged cells associated with the periphery, suggesting a weak propensity for heterotypic interactions (Fig. 6C,D), and (iii) clumps containing both green and red fluorescent cells suggesting the occurrence of heterotypic interactions (Fig. 6E,F).

**Figure 7:**
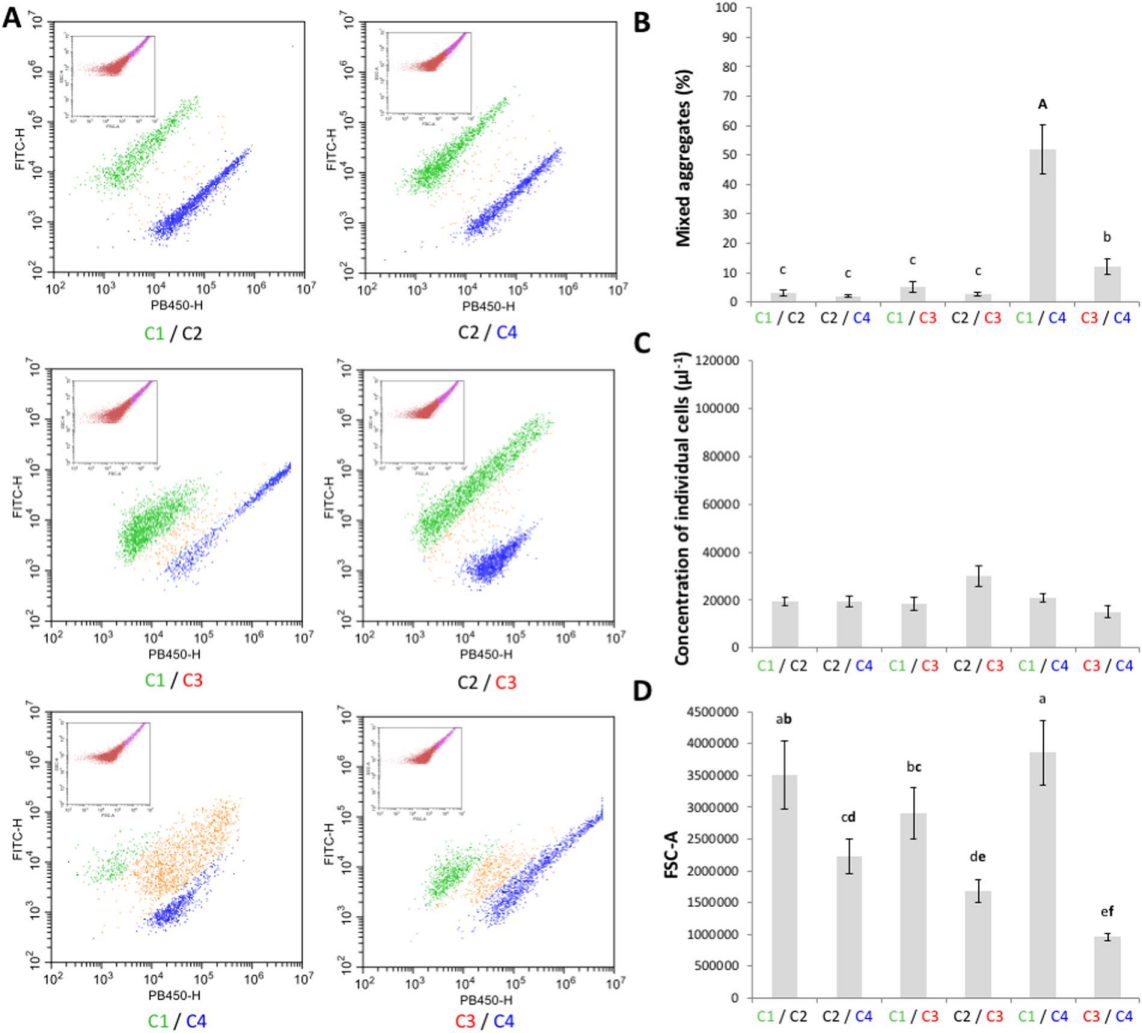
Characterisation of heterotypic interactions of Ag43 variants by flow cytometry. Flow cytometry analyses were performed by mixing SRUD-Green and SRUD-Blue expressing respectively Ag43^C1-PT^ (C1) and Ag43^C2-PT^ (C2), Ag43^C2-PT^ and Ag43^C4-PT^ (C4), Ag43^C1-PT^ and Ag43^C3-PT^ (C3), Ag43^C2-PT^ and Ag43^C3-PT^, Ag43^C1-PT^ and Ag43^C4-PT^, or Ag43^C3-PT^ and Ag43^C4-PT^. (**A**) Based on the SSC-A over FSC-A representation (pictured in the insert), the fluorescent FITC-H over PB450-H representation focused on the gate corresponding to the cell aggregates as previously defined (Supplementary Fig. S3). Graphs correspond to one representative experiment. (**B**) The percentage of mixed cell aggregates was calculated from the events detected in orange cloud over the total number of events corresponding to bacterial cell aggregates. (**C**) The concentration of single individual cells was calculated from the corresponding gate as previously defined (Supplementary Fig. S3). (**D**) The size of the aggregates was recovered from the gate for cell aggregates as previously defined (Supplementary Fig. S3).

Flow cytometry analyses confirmed the absence of heterotypic interaction between cells expressing Ag43^C1-PT^ and Ag43^C2-PT^ or cells expressing Ag43^C2-PT^ and Ag43^C4-PT^ (Fig. 7A) as the percentage of mixed aggregates were systematically estimated at less than 3% (Fig. 7B). Similarly, flow cytometry analyses confirmed the absence of significant heterotypic interactions as observed by epifluorescent microscopy for combinations of cells expressing Ag43^C1-PT^/Ag43^C3-PT^ or Ag43^C2-PT^/Ag43^C3-PT^, with a percentage of mixed aggregates estimated at less than 5% (Fig. 7A,B). However, combinations of cells expressing Ag43^C1-PT^/Ag43^C4-PT^ or Ag43^C3-PT^/Ag43^C4-PT^ showed significant heterotypic interactions with mixed cell aggregates estimated at just above 50% and 12%, respectively (Fig. 7A,B). In both cases, the percentage of mixed cell aggregates were significantly lower than the corresponding homotypic combinations (Fig. 5B). For all heterotypic combinations, no significant differences could be observed in the concentration of single cells (Fig. 7C). Heterotypic combinations resulted in aggregates with different sizes, some showing a mean size significantly larger than the corresponding homotypic combinations (i.e. Ag43^C1-PT^/Ag43^C2-PT^, Ag43^C1-PT^/Ag43^C3-PT^, and Ag43^C1-PT^/Ag43^C4-PT^) (Figs 5D and 7D). Interestingly, the association of cells expressing Ag43^C3-PT^ with cells expressing any other class of Ag43 resulted in significantly lower concentration of single individual cells (Figs 5C and 7C) as well as significantly larger cell aggregates (Figs 5D and 7D) when compared to homotypic interactions.

Overall, heterotypic interactions between Ag43 from different classes is rather compromised, since no heterotypic interactions were observed in four out of the six possible combinations of prototypical Ag43 from C1 to C4. When heterotypic interactions occurred, the percentages of mixed aggregates were significantly lower than for homotypic interactions. Nonetheless, the highest percentage of mixed aggregates resulted from cells expressing Ag43^C1-PT^/Ag43^C4-PT^, both passengers consisting of the SL1 subdomain (Fig. 2B). Conversely, and despite exhibiting the same SL2 subdomain, no heterotypic interactions could be observed for cells expressing Ag43^C2-PT^/Ag43^C3-PT^. Surprisingly, some heterotypic interactions were observed between cells expressing Ag43^C3-PT^/Ag43^C4-PT^, which would involve interactions between SL1 and SL2 subdomains.

### Ag43-mediated bacterial autoaggregation involves both specific and nonspecific interactions

**Figure 8:**
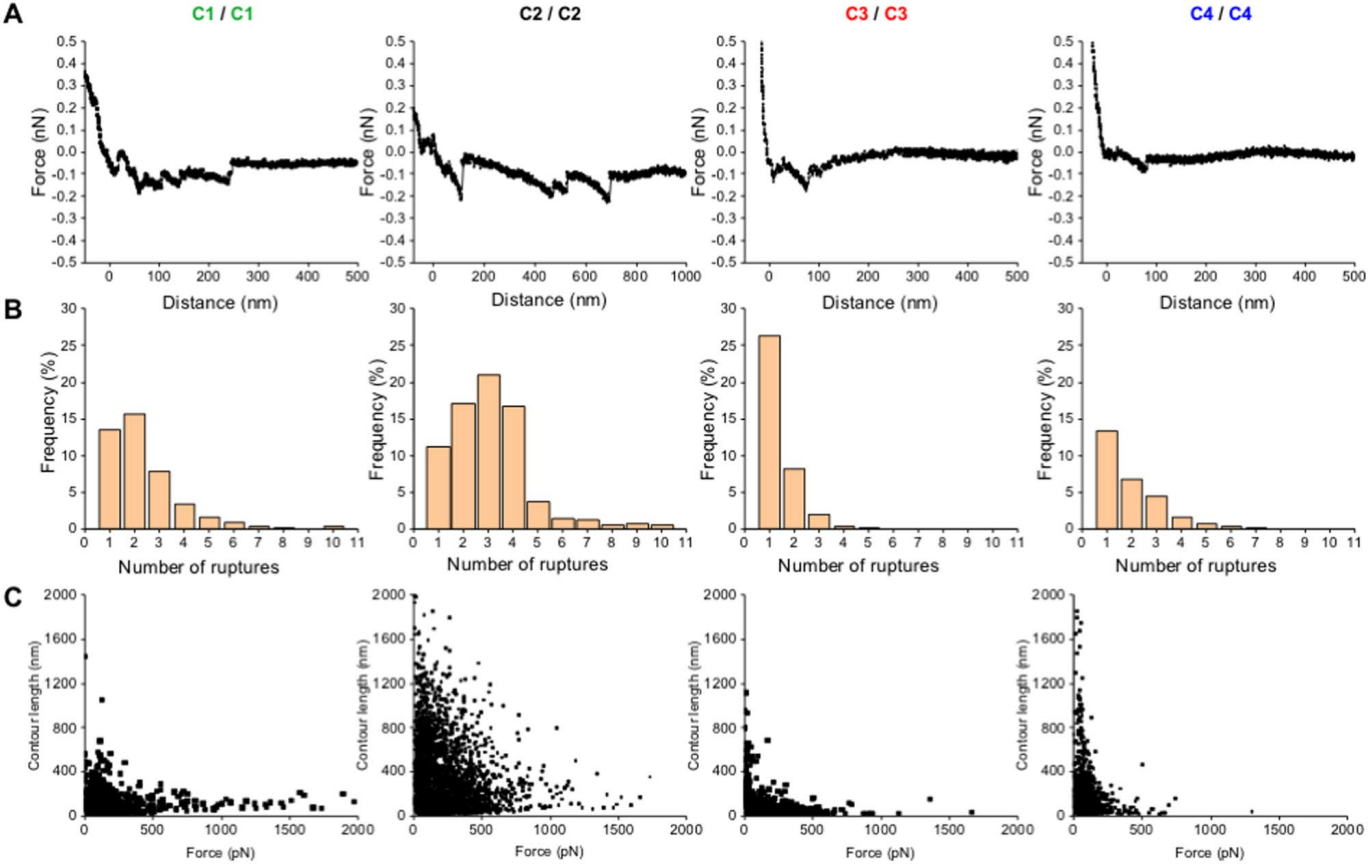
Retraction force curve and analysis of the rupture events during homotypic interactions of *E*. *coli* cells expressing different Ag43 variants. (**A**) Retraction force curves. (**B**) Rupture events measured per force curve. (**C**) Distribution of adhesion force measured between two *E*. *coli* cells expressing different Ag43 as function of the contour length for each force-distance curves. Analyses were performed using SRUD expressing the Ag43 from the different classes, namely Ag43^C1-PT^ (C1), Ag43^C2-PT^ (C2), Ag43^C3-PT^ (C3) or Ag43^C4-PT^ (C4).

Our data suggest the Ag43-Ag43 interactions could be stronger or weaker for some pairings. To quantify this and reinforce the hypothesis, single cell-force spectroscopy experiments were carried out to gain further insight in the adhesive forces at play in the autoaggregation process. First, the self-interaction between cells expressing different Ag43 variants was investigated by recording multiple force curves to measure the frequencies of adhesive events and adhesion forces between two *E*. *coli* cells (Table 2). For homotypic interactions, Ag43^C2-PT^ expressing cells exhibited the highest percentage of adhesive events, whereas bacterial cells expressing Ag43^C3-PT^ exhibited the lowest (approximately 72 and 28%, respectively). For homotypic associations of Ag43^C1-PT^ cells, the distribution of the adhesion forces show the highest most probable rupture force with 113 pN (Table 2 and Supplementary Fig. S6). Interestingly, adhesion forces for Ag43^C2-PT^ homotypic interactions showed a bimodal distribution with two maxima at 91 and 223 pN, respectively, which suggest that two different interaction conformations can exist for this protein (Table 2 and Supplementary Fig. S6). Force curves show that multiple adhesion events took place during the self-interaction of Ag43^C1-PT^ or Ag43^C2-PT^ expressing cells, whereas single interaction events were mostly recorded for Ag43^C3-PT^ or Ag43^C4-PT^ self-interaction (Fig. 8A). For cells expressing Ag43^C1-PT^ or Ag43^C2-PT^ but not Ag43^C3-PT^ or Ag43^C4-PT^, statistical analysis of the number of rupture events detected per force curves evidenced a maximum of 10 rupture events, indicating sequential unfolding of these homotypic interactions (Fig. 8B). Using the WLC (worm-like chain) model (allowing to measure the contour length of the unfolded molecule, which is defined as the length of the fully extended protein), the distribution of adhesion forces as a function of the contour length of unfolded Ag43 displayed different type of interactions (Fig. 8C). For Ag43^C2-PT^, Ag43^C3-PT^ and Ag43^C4-PT^, the presence of adhesive events associated with unfolding at contour length of >400 nm and rupture forces (50–200 pN) suggests the presence of specific interactions between two cells via multiple electrostatic and/or hydrogen bonds. At the same time, adhesive events with contour length of <100 nm were observed for all Ag43 variants and suggests non-specific hydrophobic interactions. Taking together, these observations could explain the highest frequency of adhesive events measured for Ag43^C2-PT^.

**Table 2:** Percentage of adhesive events and values of the rupture force measured by single-cell force spectroscopy from bacteria expressing different Ag43 variants.

Binding Couple^A^	Adhesive Events (%)^B^	Rupture Force (PN)^C^
Homotypic Interactions		
C1/C1	44.0 ± 9.0	113 ± 13
C2/C2	71.9 ± 14.2	91 ± 30; 223 ± 175
cC3/C3	27.8 ± 1.5	78 ± 16
C4/C4	37.3 ± 10.3	84 ± 108
Heterotypic Interactions		
C4/C2	4.2 ± 2.6	149 ± 90
C4/c1	73.8 ± 6.1	67 ± 30; 350 ± 200
C4/c3	31.9 ± 6.2	68 ± 40; 315 ± 100

^A^*E*. *Coli* SRUD was used to express the prototypical Ag43 variants from the different classes, namely Ag43^C1-PT^ (C1), Ag43^C2-PT^ (C2), Ag43^C3-PT^ (C3) and Ag43^C4-PT^ (C4).

^B^Data correspond to mean value ± standard deviation and were extracted from AFM force-distance curves analysed with WLC model as described in the Methods section (Figs S4 and S5).

^C^Data correspond to the most probable rupture force (most frequent and maximum value) ± the range of the distribution of the adhesion forces and were extracted from AFM force-distance curves analysed with WLC model as described in the Methods section (Figs S4 and S5). From bimodal distributions, two maxima could be calculated.

**Figure 9:**
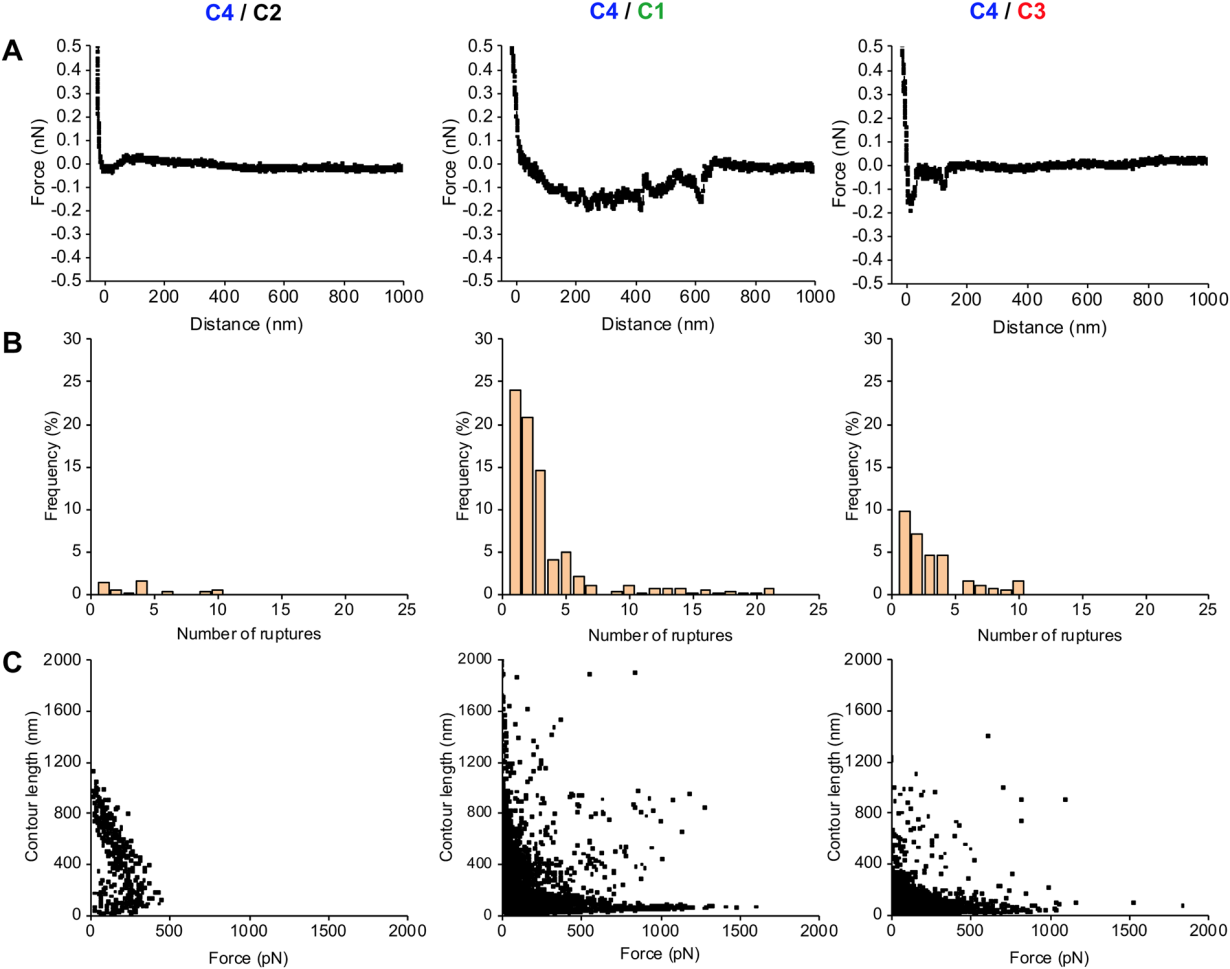
Retraction force curve and analysis of the rupture events during heterotypic interactions of *E*. *coli* cells expressing different Ag43 variants. (**A**) Retraction force curves. (**B**) Rupture events measured per force curve. (**C**) Distribution of adhesion force measured between two *E*. *coli* cells expressing different Ag43 as function of the contour length for each force-distance curves. Analyses were performed by mixing SRUD expressing Ag43 from different classes, respectively Ag43^C1-PT^ (C1), Ag43^C2-PT^ (C2), Ag43^C3-PT^ (C3) or Ag43^C4-PT^ (C4).

As mixed cell aggregates were observed between cells expressing Ag43^C4-PT^ and (i) Ag43^C1-PT^, as well as at lesser extent with (ii) Ag43^C3-PT^, but not with (iii) Ag43^C2-PT^, these heterotypic interactions were further investigated. A high frequency of adhesive events reaching approximately 74% was recorded between cells expressing Ag43^C4-PT^ and Ag43^C1-PT^ (Table 2). Conversely, this frequency remained moderate between cells expressing Ag43^C4-PT^ and Ag43^C3-PT^ (approximately 32%) and as expected extremely poor between Ag43^C4-PT^ and Ag43^C2-PT^ (approximately 4%), the latter indicative of an absence of significant interactions. Interestingly, the distribution of adhesion forces in heterotypic interactions showed two maxima corresponding to a bimodal distribution indicating the presence of two Ag43 binding conformations (Supplementary Fig. S7). For heterotypic interactions between cells expressing Ag43^C4-PT^ and Ag43^C1-PT^, multiple rupture events were observed with a maximum of 21 rupture events (Fig. 9A,B). Additionally, Ag43^C1-PT^ and Ag43^C4-PT^ heterotypic association seemed to also exhibit both non-specific hydrophobic and several specific interactions via multiple electrostatic and/or hydrogen bonds (Fig. 9C). In summary, whenever the occurrence of homotypic or heterotypic interactions, AFM analyses emphasised the important role of both specific and nonspecific interactions in the Ag43-mediated bacterial autoaggregation.

## Discussion

Previous phylogenetic analysis had simply divided Ag43 homologues into two distinct clusters, namely SF-I and SF-II^24^. Our further phylogenetic network analysis on the functional Ag43 passenger domains responsible for bacterial autoaggregation, has now revealed for the first time the existence of four distinct Ag43 phylogenetic classes, C1 to C4. Despite these divisions, structural modelling predicted that all Ag43 passengers form a similar L-shaped turn, the passenger structure themselves being subdivided into SL, EJ and BL subdomains. Ag43 passengers consistently displayed overall right-handed parallel β-helix fold with the exception of the two hairpins (each formed of two antiparallel β-sheets) delimiting the EJ subdomain. While there was evidence of structural variation reported in the literature^30,31^, the present investigation evidenced that each SL, EJ and BL subdomain belong to two distinct phylogenetic types, whereby combinations of SL with EJ-BL subdomains divide the four different classes of Ag43 passengers. This new finding represents a major breakthrough in the field as it provides a rational framework for directed functional investigations of Ag43.

**Figure 10:**
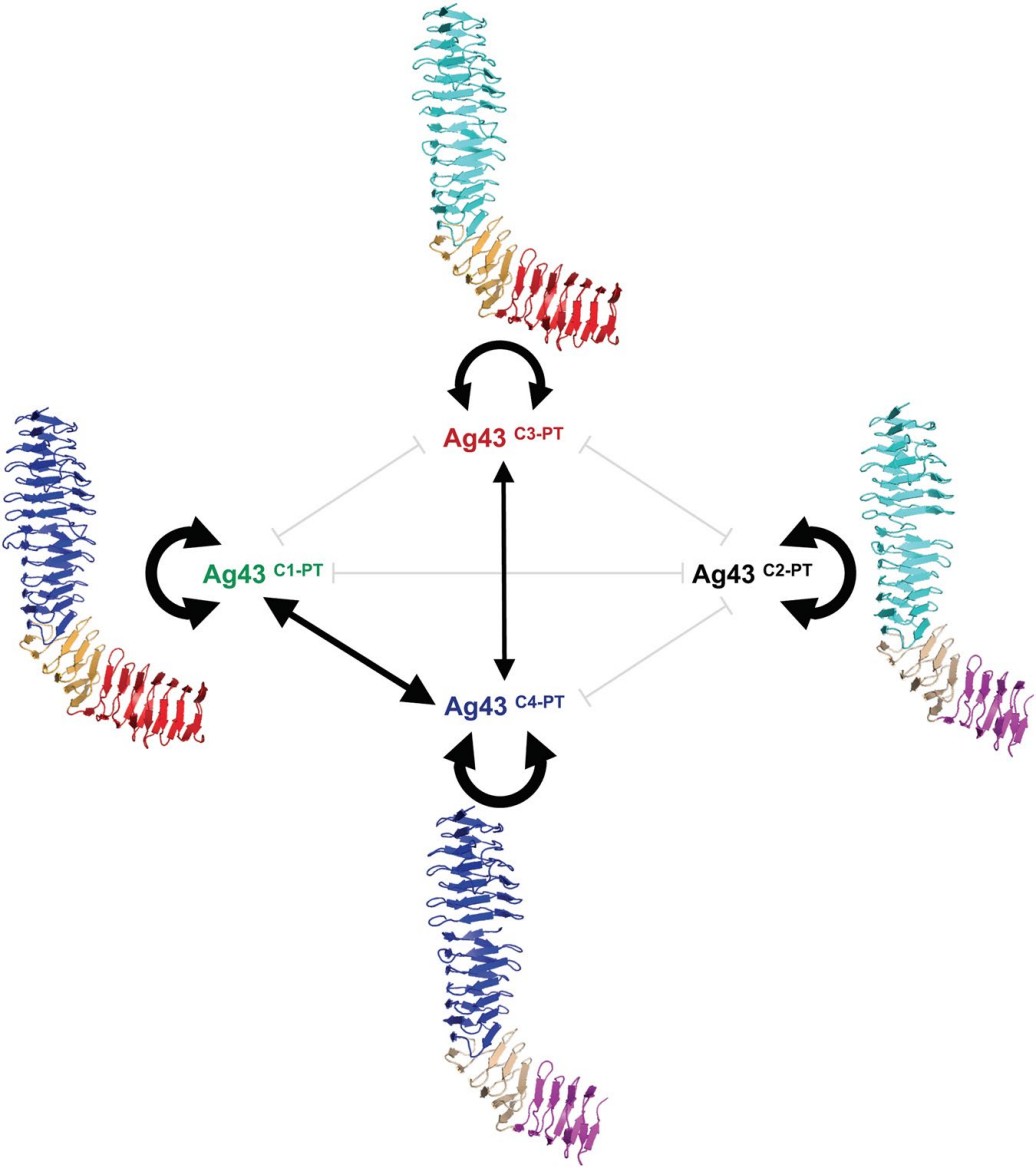
Summary of the different combinations of homotypic and heterotypic interactions of *E*. *coli* cells expressing Ag43 from the different classes in relation with the model structure of the passengers. Size of the black arrows schematically represent the force of the interactions. Grey lines indicate the tested combinations where no interaction could be experimentally evidenced. All structures of the Ag43 passengers were modelled using Phyre^54,55^.

With this new understanding of the diversity of Ag43, the autoaggregation phenotype was reinvestigated based on this updated Ag43^C1^, Ag43^C2^, Ag43^C3^ and Ag43^C4^ classification. Following bacterial cell expression of several Ag43 from each of these classes, the most striking difference was based in the significant delay of the sedimentation kinetics for Ag43 proteins belonging to the class 3, compared to the Ag43^C1^, Ag43^C2^ and Ag43^C4^. While phenotypical analyses using different Ag43 have been reported in the scientific literature^30,31^, no rationale had been previously described. Further, some contradictory results have also been conveyed^24^ that could be attributed to differences in critical parameters in the experimental set-up, such as the culture media (e.g. complex over chemically defined) or the bacterial genetic background (e.g. the use of *E*. *coli* laboratory strain for Ag43 expression still containing *agn43* and/or *fim*). Here, using the nonaggregative *E*. *coli* SRUD strain and the same growth medium, the Ag43a from UPEC CFT073 (UPI00000E4A66) appeared to exhibit a slow sedimentation as originally reported^31^. Together with Ag43b from UPEC CFT073 (UPI00000E49D2) and other Ag43^C3^, their sedimentation kinetics were significantly slower than Ag43^C1^, Ag43^C2^ or Ag43^C4^. Further, these aggregation experiments also revealed differences within C3 with Ag43a showing the strongest sedimentations properties in this class compared to other proteins like Ag43b, which showed weaker cell settling properties. Therefore, the latter Ag43 homologue better represents the phenotypical properties of this class and could be considered a prototypical Ag43^C3^. Most of the current understanding on Ag43 stems from the work done on the prototypical Ag43^C1-UPI00003B25EE^ from *E*. *coli* K12 MG1655, which has a SL1-EJ1-BL1 passenger. To date, only one article has been reported characterising Ag43^C2-UPI00000D07E5^ from EHEC O157:H7 EDL933, which presents a SL2-EJ2-BL2 passenger^26^. Our work is the first to report the characterisation of Ag43 homologues from C4, such as Ag43^C4-UPI0001E6D993^ from enterotoxigenic *E*. *coli* O78:H11 H10407, which exhibits a SL1-EJ2-BL2 passenger^32^. We show that bacterial cells expressing different Ag43 variants were able to induce autoaggregation through homotypic interactions as supported by microscopic examination and flow cytometry analyses (Fig. 10). However, differences were observed among the four Ag43 classes, with Ag43^C3-PT^ showing slower sedimentation kinetics, looser autoaggregation pellets and significantly smaller aggregates associated with lower rupture force revealed by AFM analyses. With regard to single-molecule force spectroscopy analysis, it is worth noting that for Ag43^C1-PT^ similar force curves and rupture forces of 100–200 pN were previously recorded^33^.

Considering that heterologous associations have been reported between very different members of the SAATs family, namely Ag43, AIDA-I and TibA^17,19^, the paucity of heterotypic interactions between Ag43 variants evidenced in this work was unexpected. While the recognition and involvement of binding interfaces in both homotypic and heterotypic interactions were suggested in the case of co-interacting SAATs, the restricted number of binding pairs respective to the different Ag43 variants suggest the involvement of different binding interfaces. Structural studies of homotypic interactions in Ag43^C3-UPI00000E4A66^ revealed a Velcro-like handshake mechanism via trans-association of the SL subdomains stabilised by multiple hydrogen bonds and electrostatic interactions^25^. Ag43^C1-PT^ and Ag43^C4-PT^ share the SL1 subdomain and we could observe some heterotypic interactions between cells expressing those Ag43 homologues. Nonetheless, the level and force of this heterotypic association are significantly weaker than the corresponding homotypic interactions. Differences at amino acid level between Ag43^C1-PT^ and Ag43^C4-PT^ proteins probably result in a reduced number of interactions stabilising the hetero-dimers compared to the homodimers. Additionally, the EJ-BL subdomains could also play an indirect role in the Ag43-Ag43 interaction. Indeed, previous work showed that modification of the EJ subdomain altered the angle between the SL and BL subdomains and the overall L-shaped structure, completely abolishing the ability of cells expressing Ag43 to form aggregates^25^. The absence of aggregates between cells expressing Ag43^C2-PT^ and Ag43^C3-PT^, despite both having a SL2, suggests this subdomain is less tolerant towards heterotypic associations and may be due to the binding interface regions being more selective in forming interactions. Similarly, no heterotypic interactions could be detected for combinations of Ag43 exhibiting different SL subdomain, namely SL1/SL2, except in the case of cells expressing Ag43^C3-PT^ and Ag43^C4-PT^. Considering the only two observed heterotypic interactions involved Ag43^C4-PT^, it is tempting to hypothesise that the amino acid composition of SL1 and the EJ2-BL2 architecture in the passenger are more permissive to heterotypic interactions. Diverse EJ-BL subdomains may induce different bending angles to the L-shape Ag43 structure and modulate the ability to form heterologous associations. However, the molecular modelling approach used here to model the structure of different Ag43 homologues was not able to detect any differences as the angle between SL and BL biased the bending towards the Ag43^C3-UPI00000E49D2^ template. Solving the experimental structures of Ag43 belonging to classes 1, 2 and 4, as well as other SAATs is critical to further inform the role of the different passenger subdomains in inducing autoaggregation and potentially predict heterologous associations among different SAATs.

Autoaggregation is a widespread phenomenon in bacteria but its ecophysiological importance is not fully elucidated. Several direct and side effects of autoaggregation have been reported in the literature, *e*.*g*. mechanism of adaptation against different stresses such as host defences as well as promoting cell invasion and colonisation^18^. The differential autoaggregation as well as the paucity of heterotypic interactions suggests that the Ag43 variants could play a role in structuring the *E*. *coli* population in an ecosystem by rallying or excluding some bacterial strains and thus stratifying the bacterial community. Considering that up to three *agn43* alleles can be present in one bacterial genome^24^, further genomic investigations would be required to characterise the prevalence, distribution and diversity of Ag43, such as the combinations of classes and domain shuffling, among *E*. *coli* and within the Bacteria kingdom, as well the correlations with some features of the bacterial strains, such as the origin, the level of virulence or serotype. The reason why *E*. *coli* can carry different *agn43* alleles in one genome and express so many different variants of Ag43 is still unclear^24^ but their implication in social behaviour especially through selective altruism following a greenbeard effect is an attractive concept^34–36^. Considering *agn43* can identify the presence of copies of themselves in other cells through Ag43-Ag43 interactions, this might induce some kind of nepotism behaviour toward those individual cells. The diversity of Ag43 variants and multiplicity of alleles could then favour differential and beneficial interactions between individual cells in a bacterial population.

These different aspects would undoubtedly require further investigations to understand how it could contribute to colonisation process and specific adaptation of *E*. *coli* within an ecosystem, both considering commensal and pathogenic *E*. *coli* strains but also the complexity of bacterial communities, including the microbiota and the rest of the biotope as well as the biocenosis.

## Methods

### Bacterial strains and culture conditions

Experiments were carried out in an *E*. *coli* K12 MG1655 *∆fim ∆agn43* deficient in type 1 fimbriae and Ag43 expression making the cells unable to autoaggregate, hereafter referred as *E*. *coli* SRUD (in honour to the inventors Sebastian Reidl and Ulrich Dobrindt)^30^. The most relevant bacterial strains used in this study are listed in Table 1; the exhaustive list of strains and plasmids is presented in details in Supplementary Table S1. Bacteria were cultured in LB (lysogeny broth) medium^37,38^. Briefly, strains were plated on LB agar medium and incubated overnight at 37 °C and a preculture was set up from one colony in LB broth at 37 °C in an orbital shaker (150 rpm). When required, LB medium was supplemented with antibiotics, i.e. ampicillin (100 μg.ml^−1^), chloramphenicol (25 μg.ml^−1^) or kanamycin (40 μg.ml^−1^). Arabinose at a final concentration of 0.2% (w/v) was added in the medium to induce gene expression from pBAD/Myc-HisA vector (ThermoFischer Scientific).

### Cloning and expression of genes encoding Ag43 variants

CDS encoding the different Ag43 variants (namely UniParc: UPI003B25EE, UPI000224229A, UPI0001F8D949, UPI00000D07E5, UPI0001F68B55, UPI00005F0EEA, UPI00000E4A66, UPI00000E49D2, UPI0001EB6ED2, UPI000037640F, UPI0001888EFC, UPI000000CD3C, UPI0001E6D993, UPI00092D946C, UPI00092DE8CA) were synthesised *de novo* by GeneArt (ThermoFischer Scientific) and cloned into the pBAD/Myc-HisA expression vector at the XhoI and HindIII restriction sites. Plasmid constructs were transformed into *E*. *coli* SRUD by electroporation using a Gene Pulser II and Pulse Controler Plus (Bio-Rad) (12.5 kV/cm, 200Ω, 25 μF) prior to selection on LB agar supplemented with ampicillin.

To obtain fluorescent bacterial strains, genes encoding for mRuby2, mTagBFP2 and mEmerald fluorescent proteins (GeneBank: AFR60232.1, AIQ82697.1 and AIA24532.1, respectively) were synthesised by GeneArt including a constitutive promoter (iGEM: BBa-J23119) and ribosome binding site (RBS) from pBAD/Myc-HisA, prior to cloning into the pACYC184 *Ava*I and *Bsu*36I/*Tth*111I restriction sites to generate pVaAgRed, pVaAgBlue and pVaAgGreen respectively, whose p15a replication origin belongs to a different incompatibility group from the pBR322 replication origin in pBAD-vector series. Plasmid constructs were transformed by electroporation in *E*. *coli* SRUD with or without a pVaAg-vector series and selection was carried on LB agar supplemented with chloramphenicol and when required with the additional supplementation of ampicillin.

### Immunodetection and subcellular localisation of Ag43 variants

Bacterial cells were grown with arabinose till late exponential phase as described above. Cells were harvested by centrifugation (7 000 *g*, 5 min, 4 °C), washed and resuspended in PBS (0.01 M, 1 mM PMSF, pH8). Cells were disrupted with glass microbeads using FastPrep (20 s twice at 6 m.s^−1^). Part of lysed whole cells was reserved and the other part subjected to cell fractionation using a modified sarkosyl (sodium lauroyl sarcosinate) method^39,40^. Briefly, unbroken cells and cellular debris were removed by centrifugation (12 000 *g*, 10 min, 4 °C) before the supernatant was ultracentrifuged (100 000 *g*, 40 min, 4 °C). The supernatant was retained as the soluble fraction (cytoplasm and periplasm), and the pellet was resuspended in 2% sarkosyl (20 min, room temperature) followed by another ultracentrifugation. The pellet corresponded to the sarcosyl-insoluble fraction gathering the OM. Cold acetone was added to the supernatant (overnight at −20 °C), centrifuged and the pellet corresponding to the sarcosyl-soluble fraction gathering the IM. Protein concentrations were determined using the Bradford method^41^.

Proteins from the different fractions were separated by SDS-PAGE. Cell fractions were diluted in Laemmli buffer and heated up at 95 °C for 5 min. From prior testing with increasing amounts of whole cell fractions, 1 µg of total proteins was determined as a non-saturating concentration and equal amounts of each fraction based on the starting material were loaded per lane using Mini-PROTEAN Precast TGX Gels 4–15% (Bio-Rad). Precision Plus Protein Dual Color Standards (Bio-Rad) was used to enable molecular mass estimation as well as transfer control. For Western blotting, proteins were dry transferred from SDS-PAGE on iBlot Transfer Stacks using iBlot 2 Dry Blotting System (Invitrogen). After saturating unoccupied protein-binding sites with blocking agents, membranes were probed with specific anti-Ag43 primary antibodies (dilution 1/5000 in PBS with 0.1% tween and 0.1% milk, 1 h, room temperature). Rabbit Ag43 antibodies were obtained as previously described^42,43^ and are crossed reactive between Ag43 variants^44^. Goat anti-Rabbit IgG antibodies conjugated with horseradish peroxidase was used as secondary antibodies (dilution of 1/10 000, 1 h, room temperature) and TMB (3,3′,5,5′-tetramethylbenzidine) was used to detect the enzymatic activity. Enzymatic reaction was stopped by rinsing the membranes with milliQ water. Western blots were imaged under white light conversion screen using E-BOX (VILBER).

### Phylogenetic analysis of protein sequences

The Ag43 protein sequences were retrieved from the literature^24,28^ and the corresponding passengers recovered by removing the SP, AC1 and translocator regions. For identification of the SPs in Ag43 proteins, the sequences submitted to SignalP v4.1^45^ were deleted from the ESPR (InterPro: IPR024973), which was first identified using InterProScan v5.22^46^. The AC1 domains were identified based on a structural alignment as previously described^3^. The translocators were identified using the twin HMM (Hidden Markov Model) strategy for AT detection^47^. Finally, truncated and redundant Ag43 passenger sequences were removed, resulting in a dataset of 106 distinct Ag43 passengers (Supplementary Table S1). Then, the amino acid sequences were aligned with T-Coffee in the Expresso mode for automatic incorporation of structural information^48,49^ using the solved structure of Ag43 (PDB: 4KH3) as template. The relatedness among protein sequences was studied using a Neighbor-Net phylogenetic network approach with SplitsTree v4.14.8 using the Hamming uncorrected-P distance^50^. The most robust splits of the network were identified by bootstrap using a 95% threshold over 1,000 pseudo-replicates. A similar phylogenetic analysis was repeated for the different passenger subdomains. Pairwise sequence similarities and identities were obtained using Needleman-Wunsch alignment of two sequences with BLAST^51,52^ (Supplementary Table S1).

### Identification of conserved motifs and structural modelling

Conserved motifs were searched using InterProScan v5.22^46^ and interrogating InterPro (IPR) v61.0 database^53^. Three-dimensional structures were modelled with Phyre v2.0^54,55^ as well as I-TASSER v5.1^56,57^. The types of β-solenoid motif present in the β-helices were predicted using PfScan^29^. In β-helices, rungs were defined as adjacent parallel β-strands. For selected variants as presented in Fig. 2, Ag43 homolog models were checked and modified in the program COOT^58^ using its Ramachandran, geometry and rotamer analysis validation software. The quality of the models were then assessed using ProCheck^59^ with its Ramachandran errors reported as percentages of generously allowed and disallowed amino acids (Supplementary Table S3). Protein structures were visualised using PyMOL v1.7.4^60,61^.

### Autoaggregation assay

Autoaggregation assays were performed as previously described^62^. Briefly, overnight precultures were diluted 1:100 and grown in LB at 37 °C under orbital shaking. Arabinose was added in early exponential phase (OD_600 nm_ = 0.25) to induce the Ag43 expression from pBAD-vector series (Supplementary Table S2). Cell suspensions were placed in conical tubes at the same OD_600 nm_, incubated statically and sampled every hour from the top of the tube to measure the OD_600 nm_. Observations in phase-contrast microscopy were made at the end of the experiments from sediment at the bottom of the tube to confirm the bacterial cell autoaggregation.

### Image acquisition and analysis of phase contrast and epifluorescence microscopic observations

Microscopic observations of bacterial cells were performed in phase contrast and phase contrast epifluorescence reflected light. Image acquisition was performed using an Axioplan 2 microscope coupled to an AxioCam MRm camera driven by the AxioVision 4.8 software (Carl Zeiss). For phase contrast microscopy, a 40 × /0.75 objective (Plan-NEOFLUAR) was used and the fluorescence light source was a mercury lamp (HBO100W/2, AttoArc, Carl Zeiss). For fluorescence image acquisition a SYTO9-AO-FITC cube was fitted with a 450–490 nm exciter, 515–565 nm emitter, and 510 nm dichroic mirror as well as a Propidium iodide-Rhodamine (Con A) cube containing 546/12 nm exciter, 590 nm emitter, and 580 nm dichroic mirror (Carl Zeiss).

### Quantification of bacterial cell interactions by flow cytometry

To investigate interactions of bacterial cells expressing Ag43 variants, *E*. *coli* SRUD-Green (carrying pVaAgGreen) or *E*. *coli* SRUD-Blue (carrying pVagAgBlue) were transformed with pBAD/Myc-HisA vectors encoding the different Ag43 variants or empty vector (negative control with non-aggregative *E*. *coli* SRUD bacterial cells) (Supplementary Table S2). Overnight *E*. *coli* SRUD-Green and *E*. *coli* SRUD-Blue precultures were diluted 1:100 and grown as described above in LB until early exponential phase. At this point, samples from these two different bacterial cultures were adjusted at an OD_600 nm_ of 0.25, mixed together at a 50/50 (v/v) ratio with arabinose and grown at 37 °C under shaking until an OD_600 nm_ of 0.5. Conical tubes were then incubated statically for 6 h and bacterial cells fixed with paraformaldehyde (2% final concentration) for 15 min. Cells were collected in the bottom of each tube and diluted in PBS (phosphate-buffered saline).

Samples were filtered on a 40-μm-nylon cell strainer prior to flow cytometry analysis (CytoFLEX, Beckman Coulter). FSC (forward scatter), SSC (side scatter), FITC (fluorescein isothiocyanate; 488 nm exciter, 525/540 nm emitter) and PB450 (Pacific Blue 450 nm; 405 nm exciter, 450/445 nm emitter) axes were set to log display. Areas for FSC and SSC (FSC-A and SSC-A, respectively) were used to discriminate aggregates from single individual cells, whereas heights for FITC and PB450 (FITC-H and PB450-H, respectively) were used to quantify co-interactions within aggregates (Supplementary Fig. S3). At least 3000 events/sec were recorded for each sample and data were analysed with CytExpert software v2.2 (Beckman Coulter). Results were expressed as percentages of mixed aggregates over the total aggregates.

### Atomic force microscopy analyses

For cell probe preparation, bacterial cells were handled as for the autoaggregation assay prior to dilution in PBS buffer to an OD_600 mn_ of 0.2. One mL of bacterial suspension was directly deposited onto glass slide pre-coated with polyethylenimin (PEI) as previously described^63^. Briefly, cleaned glass slides are immersed in 0.2% PEI solution for 1 h, then extensively rinsed with Milli-Q water and finally dried under nitrogen) for 30 min and then the surface was rinsed twice with PBS buffer to get rid of un-adhered free bacteria and remaining residues from growth medium.

A Nanowizard III AFM (JPK-Bruker) was used to attach to atipless silicon nitride cantilevers (NP-010, Bruker) 6 µm borosilicate microspheres using UV curing resin (Norland optical adhesive 63 Glue, Norland Edmund Optics). Sphere attached cantilevers were cured under UV light (λ = 400 nm) for 3 min. The cantilever was then immersed for 1 h in a 0.2% PEI solution and washed. Functionalised cantilevers were calibrated before analysis by the thermal noise method^64^ and spring constants were found to be 0.048 ± 0.011 N/m. Colloidal probe^65^ was brought into contact with an isolated cell for 3 min, and the obtained cell probe was then moved to another cell to perform single cell-force spectroscopy measurements. Suitable parameters for cell attachment are a contact force of 1 nN, a contact time of 2 s and an approach and retract rate of 1 µms^−1^.

Single cell-force spectroscopy experiments were carried out with a Nanowizard III atomic force microscope (JPK-Bruker). Measurements were performed at room temperature in PBS buffer at pH 7.2. Force-distance curves were recorded on different cells, using a maximum applied force of 1 nN and a constant approach and retraction speed of 1 µm.s^−1^. The time of contact was set at 2 s to enhance specific interactions as described previously^66,67^. The type of interactions, *i*.*e*. specific or non-specific, could be deduced from the patterns of the force-distance curves^68–70^.

The obtained force versus distance curves were analysed with the JPK Data Processing software on the basis of the worm-like chain (WLC) model, which is suitable to describe the extension of protein. The extension *x* of the molecule versus the pulling force (or adhesion force) is given by the following equation:

$$F(x)=\,\frac{{k}_{B}T}{{l}_{p}}\,[\frac{1}{4}\,{(1-\frac{x}{{L}_{c}})}^{-2}+\,\frac{x}{{L}_{c}}-\,\frac{1}{4}]$$

where k_B_ is the Boltzmann constant, T the absolute temperature, l_p_ is the persistent length and L_c_ is the contour length. The persistent length gives information about the degree of structural rigidity of the polymer chain and is defined as the longest segment below which the chain can be considered as a rigid rod, the extension of the polymer until the point at which the force necessary to extend further and in our case to break the interaction with the functionalised AFM tip corresponds to the contour length.

Adhesion peaks were generated using Origin 8 software. Statistical differences of AFM data were assessed by analysis of the variance (one-way ANOVA test) using Origin 8. To check the specificity of the measured adhesion forces and avoid artifacts that can be associated with cell probe preparation, control experiments were performed. Use of PEI-coated probes without bacterial cell interacting on PEI-coated glass slide led to a dramatic decrease of the adhesion frequency with only 3% of the force curves presenting an adhesion peak.

### Statistical analysis

Statistical analyses were performed using GraphPad Prism v6.01. At least five biological replicates were carried out for all statistical analyses. Error bars on the figures represent the 95% confidence interval for the mean of the independent experiments. Data were statistically analysed following ANOVA one way or two way test, followed by the post-hoc Tukey test, with differences considered significant (*p* < 0.05, * or lowercase letter), very significant (*p* < 0.01, ** or bold lowercase letter), highly significant (*p* < 0.001, *** or capital letter) or very highly significant (*p* < 0.0001, **** or bold capital letter).

## Supplementary information

Supplementary Information

Table S1

Figure S1

Figure S2

## Data Availability

All data generated or analysed during this study are included in this published article and its Supplementary Material files. Other data are available from the corresponding author upon reasonable request.
