# Supplementary material for: Differential homotypic and heterotypic interactions of antigen 43 (Ag43) variants in autotransporter-mediated bacterial autoaggregation
Source: Sci Rep. 2019 Jul 31;9:11100. doi: 10.1038/s41598-019-47608-4 (PMC6668479; doi:10.1038/s41598-019-47608-4)
Supplement: Supplementary file 1 — Supplementary Information [file 41598_2019_47608_MOESM1_ESM.pdf]

SUPPLEMENTARY INFORMATION

Differential homotypic and heterotypic interactions of antigen 43 (Ag43) variants in autotransporter-mediated bacterial autoaggregation

Valentin AGEORGES [1], Marion SCHIAVONE [2,3], Grégory JUBELIN [1], Nelly CACCIA [1], Philippe RUIZ [1], Ingrid CHAFSEY [1], Xavier BAILLY [5], Etienne DAGUE [4], Sabine LEROY [1], Jason PAXMAN [6], Begoña HERAS [6], Frédérique CHAUCHEYRAS-DURAND [1,2], Amanda E. ROSSITER [7], Ian R. HENDERSON [7,8] and Mickaël DESVAUX [1,*]

[1] Université Clermont Auvergne, INRA, UMR454 MEDiS, 63000 Clermont-Ferrand, France

[2] Lallemand Animal Nutrition, 31702 Blagnac Cedex, France.

[3] LISBP, Université de Toulouse, CNRS UMR5504, INRA UMR792, INSA, 31077 Toulouse Cedex, France.

[4] LAAS-CNRS, Université de Toulouse, 31077 Toulouse Cedex, France.

[5] INRA UR346 Epidémiologie Animale, 63122 Saint Genès Champanelle, France.

[6] Department of Biochemistry and Genetics, La Trobe Institute for Molecular Science, La Trobe University, Bundoora, Victoria 3086, Australia.

[7] Institute of Microbiology and Infection, University of Birmingham, B152TT Birmingham, United Kingdom.

[8] Institute for Molecular Biosciences, University of Queensland, St. Lucia, Queensland 4067, Australia.

Table S1: Dataset of 106 Ag43 passengers. Provided as an independent Excel file.

Table S2: Complete list of bacterial strains and plasmids used in this study.

Table S3: Quality check of the Ag43 structural models.

Figure S1: Phylogenetic network trees of Ag43 passengers as well as SL, EJ and BL subdomains. Provided as independent scalable Nexus files. Network trees were generated using SplitsTree including the 106-entries dataset sequences without applying bootstrapping and confidence threshold and A: Tree of the Ag43 passengers. B: Tree of the SL subdomain. C: Tree of the EJ subdomain. D: Tree of the BL. The scale bars represent the number of substitutions per site.

Figure S2: Structure models of Ag43 passengers belonging to C1, C2, C3 and C4. Provided as independent PDB files. Three-dimensional structures of Ag43 presented in Figure 2B were modelled with Phyre, checked and modified with COOT.

Figure S3: Flow cytometry analysis of fluorescent-tagged bacterial cell aggregates. A: SRUD pBAD was used to define events corresponding to individual single cells (red gate) on a SSC-A over FSC-A representation. B: SRUD Ag43 $^{C1\text{-}PT}$ were used to determine events corresponding to bacterial cell aggregates (pink gate) versus individual single cells (red gate) on a SSC-A over FSC-A representation. C: SSC-A over FSC-A representation using *E. coli* SRUD-Green pBAD and SRUD-Blue pBAD indicating the presence of individual single cells (red gate) and the absence of co-interaction as defined above with SRUD pBAD (A). D: Focusing on the gate corresponding to the individual single cells as defined above (C), fluorescent FITC-H over PB450-H representation using SRUD-Green pBAD and SRUD-Blue pBAD allowed discriminating the individual single cells of SRUD-Green pBAD (green gate) and of SRUD-Blue pBAD (blue gate); black dots would correspond to event where two green and blue cells passed simultaneously (representing less than 5% of total events). E: SSC-A over FSC-A representation using *E. coli* SRUD-Green Ag43 $^{C1\text{-}PT}$ and SRUD-Blue Ag43 $^{C1\text{-}PT}$ indicating the formation of bacterial cell aggregates (pink gate) as defined above with

SRUD Ag43 $^{C1-PT}$ (B). F: Focusing on the gate corresponding to the bacterial cell aggregates defined above (E), fluorescent FITC-H over PB450-H representation using SRUD-Green Ag43 $^{C1-PT}$ and SRUD-Blue Ag43 $^{C1-PT}$ allowed to discriminate the aggregates formed by SRUD-Green Ag43 $^{C1-PT}$ (green gate), the aggregates formed by SRUD-Blue Ag43 $^{C1-PT}$ (blue gate) and the mixed-aggregates formed by SRUD-Green Ag43 $^{C1-PT}$/ SRUD-Blue Ag43 $^{C1-PT}$ (orange gate) here indicative of homotypic interactions (referring to interactions between identical Ag43).

Figure S4: Flow cytometry analyses of co-cultures of nonaggregative with aggregative fluorescent-tagged bacterial cells. Flow cytometry analyses were performed by mixing nonaggregative (NA) SRUD-Green pBAD and SRUD-Blue expressing different Ag43 variants, namely Ag43 $^{C1-PT}$ (C1), Ag43 $^{C2-PT}$ (C2), Ag43 $^{C3-PT}$ (C3) or Ag43 $^{C4-PT}$ (C4). A: SSC-A over FSC-A representation indicated the formation of bacterial cell aggregates (pink cloud). Events corresponding to single cells (red cloud) and bacterial cell aggregates were selected using gates previously defined in control experiments (Figure S3). B: Focusing on the gate corresponding to the bacterial cell aggregates, fluorescent FITC-H over PB450-H representation indicated the presence of aggregates only for cells expressing Ag43 variants (blue cloud).

Figure S5: Immunodetection and subcellular localisation of Ag43 by Western blot analyses. A: Expression level of Ag43 from whole cell extracts (1 µg of total protein per lane); Lane 1: *E. coli* SRUD pBAD, Lane 2: *E. coli* SRUD Ag43 $^{C1-PT}$, Lane 3: *E. coli* SRUD Ag43 $^{C2-PT}$, Lane 4: *E. coli* SRUD Ag43 $^{C3-PT}$, Lane 5: *E. coli* SRUD Ag43 $^{C4-PT}$. Subcellular localisation of Ag43 $^{C1-PT}$ (B), Ag43 $^{C2-PT}$ (C), Ag43 $^{C3-PT}$ (D) and Ag43 $^{C4-PT}$ (E) in *E. coli* SRUD determined through immunodetection in different cell compartments, *i.e.* whole cell (lane 1), OM (lane 2), IM (lane 3) and soluble (lane 4) fractions. MW: molecular weight marker (KDa).

Figure S6: Measurement of adhesion forces for homotypic interactions of bacterial cells expressing different Ag43 variants by AFM. Analyses were performed using SRUD expressing the

Ag43 from the different classes, namely Ag43 $^{C1\text{-}PT}$ (C1), Ag43 $^{C2\text{-}PT}$ (C2), Ag43 $^{C3\text{-}PT}$ (C3) or Ag43 $^{C4\text{-}PT}$ (C4). n.a.: non adhesive events.

Figure S7: Measurement of adhesion forces for heterotypic interactions of bacterial cells expressing different Ag43 variants by AFM. Analyses were performed by mixing SRUD expressing Ag43 from different classes, respectively Ag43 $^{C1\text{-}PT}$ (C1), Ag43 $^{C2\text{-}PT}$ (C2), Ag43 $^{C3\text{-}PT}$ (C3) or Ag43 $^{C4\text{-}PT}$ (C4). n.a.: non adhesive events.

Table S2: Complete list of bacterial strains and plasmids used in this study

Bacterial strain/plasmid	Relevant genotype and/or phenotype	References [a]
E. coli strains		
SRUD	*E. coli* K12 M C1655 Δ*fim* Δ*flu*::*kan*	1
SRUD pBAD	Empty pBAD/Myc-HisA vector in *E. coli* SRUD	This study
SRUD Ag43 $^{\text{C1-UPI00003B25EE}}$	pBAD-Ag43 $^{\text{C1-UPI00003B25EE}}$ in *E. coli* SRUD	This study
SRUD Ag43 $^{\text{C1-UPI000224229A}}$	pBAD-Ag43 $^{\text{C1-UPI000224229A}}$ in *E. coli* SRUD	This study
SRUD Ag43 $^{\text{C1-UPI0001F8D949}}$	pBAD-Ag43 $^{\text{C1-UPI0001F8D949}}$ in *E. coli* SRUD	This study
SRUD Ag43 $^{\text{C2-UPI00000D07E5}}$	pBAD-Ag43 $^{\text{C2-UPI00000D07E5}}$ in *E. coli* SRUD	This study
SRUD Ag43 $^{\text{C2-UPI0001F68B55}}$	pBAD-Ag43 $^{\text{C2-UPI0001F68B55}}$ in *E. coli* SRUD	This study
SRUD Ag43 $^{\text{C2-UPI00005F0EEA}}$	pBAD-Ag43 $^{\text{C2-UPI00005F0EEA}}$ in *E. coli* SRUD	This study
SRUD Ag43 $^{\text{C3-UPI00000E4A66}}$	pBAD-Ag43 $^{\text{C3-UPI00000E4A66}}$ in *E. coli* SRUD	This study
SRUD Ag43 $^{\text{C3-UPI0001EB6ED2}}$	pBAD-Ag43 $^{\text{C3-UPI0001EB6ED2}}$ in *E. coli* SRUD	This study
SRUD Ag43 $^{\text{C3-UPI00000E49D2}}$	pBAD-Ag43 $^{\text{C3-UPI00000E49D2}}$ in *E. coli* SRUD	This study
SRUD Ag43 $^{\text{C3-UPI000037640F}}$	pBAD- Ag43 $^{\text{C3-UPI000037640F}}$ in *E. coli* SRUD	This study
SRUD Ag43 $^{\text{C3-UPI0001888EFC}}$	pBAD- Ag43 $^{\text{C3-UPI0001888EFC}}$ in *E. coli* SRUD	This study
SRUD Ag43 $^{\text{C3-UPI000000CD3C}}$	pBAD- Ag43 $^{\text{C3-UPI000000CD3C}}$ in *E. coli* SRUD	This study
SRUD Ag43 $^{\text{C4-UPI0001E6D993}}$	pBAD-Ag43 $^{\text{C4-UPI0001E6D993}}$ in *E. coli* SRUD	This study
SRUD Ag43 $^{\text{C4-UPI00092D946C}}$	pBAD-Ag43 $^{\text{C4-UPI00092D946C}}$ in *E. coli* SRUD	This study
SRUD Ag43 $^{\text{C4-UPI00092DE8CA}}$	pBAD-Ag43 $^{\text{C4-UPI00092DE8CA}}$ in *E. coli* SRUD	This study
SRUD-Green pBAD	pVaAgGreen in *E. coli* SRUD pBAD	This study

SRUD-Red pBAD	pVaAgRed in *E. coli* SRUD pBAD	This study
SRUD-Blue pBAD	pVaAgBlue in *E. coli* SRUD pBAD	This study
SRUD-Green Ag43 $^{\text{C1-UPI00003B25EE}}$	pVaAgGreen in *E. coli* SRUD pBAD-Ag43 $^{\text{C1-UPI00003B25EE}}$	This study
SRUD-Red Ag43 $^{\text{C1-UPI00003B25EE}}$	pVaAgRed in *E. coli* SRUD pBAD-Ag43 $^{\text{C1-UPI00003B25EE}}$	This study
SRUD-Blue Ag43 $^{\text{C1-UPI00003B25EE}}$	pVaAgBlue in *E. coli* SRUD pBAD-Ag43 $^{\text{C1-UPI00003B25EE}}$	This study
SRUD-Green Ag43 $^{\text{C2-UPI00000D07E5}}$	pVaAgGreen in *E. coli* SRUD pBAD-Ag43 $^{\text{C2-UPI00000D07E5}}$	This study
SRUD-Red Ag43 $^{\text{C2-UPI00000D07E5}}$	pVaAgRed in *E. coli* SRUD pBAD-Ag43 $^{\text{C2-UPI00000D07E5}}$	This study
SRUD-Blue Ag43 $^{\text{C2-UPI00000D07E5}}$	pVaAgBlue in *E. coli* SRUD pBAD-Ag43 $^{\text{C2-UPI00000D07E5}}$	This study
SRUD-Green Ag43 $^{\text{C3-UPI00000E49D2}}$	pVaAgGreen in *E. coli* SRUD pBAD-Ag43 $^{\text{C3-UPI00000E49D2}}$	This study
SRUD-Red Ag43 $^{\text{C3-UPI00000E49D2}}$	pVaAgRed in *E. coli* SRUD pBAD-Ag43 $^{\text{C3-UPI00000E49D2}}$	This study
SRUD-Blue Ag43 $^{\text{C3-UPI00000E49D2}}$	pVaAgBlue in *E. coli* SRUD pBAD-Ag43 $^{\text{C3-UPI00000E49D2}}$	This study
SRUD-Green Ag43 $^{\text{C4-UPI0001E6D993}}$	pVaAgGreen in *E. coli* SRUD pBAD-Ag43 $^{\text{C4-UPI0001E6D993}}$	This study
SRUD-Red Ag43 $^{\text{C4-UPI0001E6D993}}$	pVaAgRed in *E. coli* SRUD pBAD-Ag43 $^{\text{C4-UPI0001E6D993}}$	This study
SRUD-Blue Ag43 $^{\text{C4-UPI0001E6D993}}$	pVaAgBlue in *E. coli* SRUD pBAD-Ag43 $^{\text{C4-UPI0001E6D993}}$	This study
Plasmids		
pBAD/Myc-HisA	Protein expression vector under the control of the inducible *ara*BAD promoter (P_{BAD}), Amp^R	Thermo Fisher Scientific
pBAD-Ag43 $^{\text{C1-UPI00003B25EE}}$	Gene encoding Ag43 $^{\text{C1-UPI00003B25EE}}$ in XhoI-HindIII digested pBAD/Myc-HisA	This study
pBAD-Ag43 $^{\text{C1-UPI000224229A}}$	Gene encoding Ag43 $^{\text{C1-UPI000224229A}}$ in XhoI-HindIII digested pBAD/Myc-HisA	This study
pBAD-Ag43 $^{\text{C1-UPI0001F8D949}}$	Gene encoding Ag43 $^{\text{C1-UPI0001F8D949}}$ in XhoI-HindIII digested pBAD/Myc-HisA	This study
pBAD-Ag43 $^{\text{C2-UPI00000D07E5}}$	Gene encoding Ag43 $^{\text{C2-UPI00000D07E5}}$ in XhoI-HindIII digested pBAD/Myc-HisA	This study
pBAD-Ag43 $^{\text{C2-UPI0001F68B55}}$	Gene encoding Ag43 $^{\text{C2-UPI0001F68B55}}$ in XhoI-HindIII digested pBAD/Myc-HisA	This study

pBAD-Ag43 $^{C2\text{-}UPI00005F0EEA}$	Gene encoding Ag43 $^{C2\text{-}UPI00005F0EEA}$ in XhoI-HindIII digested pBAD/Myc-HisA	This study
pBAD-Ag43 $^{C3\text{-}UPI00000E49D2}$	Gene encoding Ag43 $^{C3\text{-}UPI00000E49D2}$ in XhoI-HindIII digested pBAD/Myc-HisA	This study
pBAD-Ag43 $^{C3\text{-}UPI00000E4A66}$	Gene encoding Ag43 $^{C3\text{-}UPI00000E4A66}$ in XhoI-HindIII digested pBAD/Myc-HisA	This study
pBAD-Ag43 $^{C3\text{-}UPI000037640F}$	Gene encoding Ag43 $^{C3\text{-}UPI000037640F}$ in XhoI-HindIII digested pBAD/Myc-HisA	This study
pBAD-Ag43 $^{C3\text{-}UPI0001EB6ED2}$	Gene encoding Ag43 $^{C3\text{-}UPI0001EB6ED2}$ in XhoI-HindIII digested pBAD/Myc-HisA	This study
pBAD-Ag43 $^{C3\text{-}UPI0001888EFC}$	Gene encoding Ag43 $^{C3\text{-}UPI0001888EFC}$ in XhoI-HindIII digested pBAD/Myc-HisA	This study
pBAD-Ag43 $^{C3\text{-}UPI000000CD3C}$	Gene encoding Ag43 $^{C3\text{-}UPI000000CD3C}$ in XhoI-HindIII digested pBAD/Myc-HisA	This study
pBAD-Ag43 $^{C4\text{-}UPI0001E6D993}$	Gene encoding Ag43 $^{C4\text{-}UPI0001E6D993}$ in XhoI-HindIII digested pBAD/Myc-HisA	This study
pBAD-Ag43 $^{C4\text{-}UPI00092D946C}$	Gene encoding Ag43 $^{C4\text{-}UPI00092D946C}$ in XhoI-HindIII digested pBAD/Myc-HisA	This study
pBAD-Ag43 $^{C4\text{-}UPI00092DE8CA}$	Gene encoding Ag43 $^{C4\text{-} UPI00092DE8CA}$ in XhoI-HindIII digested pBAD/Myc-HisA	This study
pACYC184	Cloning vector with a p15A replication origin, Cm^R, Tc^R	2
pVaAgGreen	$P_{BBa\text{-}J23119}$::RBS^{pBAD}::*mEmerald* in AvaI-Bsu36I digested pACYC184	This study
pVaAgBlue	$P_{BBa\text{-}J23119}$::RBS^{pBAD}::*mTagBFP2* in AvaI-Bsu36I digested pACYC184	This study
pVaAgRed	$P_{BBa\text{-}J23119}$::RBS^{pBAD}::*mRuby2* in AvaI-Tth111I digested pACYC184	This study

[a] 1. Reidl, S., Lehmann, A., Schiller, R., Salam Khan, A. & Dobrindt, U. Impact of O-glycosylation on the molecular and cellular adhesion properties of the *Escherichia coli* autotransporter protein Ag43. *Int. J. Med. Microbiol*. **6**, 389-401 (2009).

2. Chang, A.C. & Cohen, S.N. Construction and characterization of amplifiable multicopy DNA cloning vehicles derived from the P15A cryptic miniplasmid. *J. Bacteriol*. **3**, 1141-1156 (1978).

Table S3: Quality check of the Ag43 structural models.

Models [a]	Generously allowed (%)	Disallowed (%)
Ag43 [C1-UPI00003B25EE (PT)]	0.5	0.0
Ag43 [C1-UPI000224229A]	0.2	0.0
Ag43 [C1-UPI000F8D949]	0.7	0.0
Ag43 [C2-UPI00000D07E5 (PT)]	1.2	0.0
Ag43 [C2-UPI0001F68B55]	0.6	0.3
Ag43 [C2-UPI00005F0EEA]	1.2	0.0
Ag43 [C3-UPI00000E49D2 (PT)]	0.5	0.0
Ag43 [C3-UPI000IEB6ED2]	0.7	0.0
Ag43 [C3-UP1000037640F]	0.2	0.0
Ag43 [C4-UPI0001E6D933 (PT)]	1.2	0.0
Ag43 [C4-UP100092D946C]	1.5	0.3
Ag43 [C4-UP100092DE8CA]	2.7	0.0

[a] The quality of the Ag43 homolog models was assessed using ProCheck. PDB files of these Ag43 structural models are provided as supplementary materials. PT: prototypical variant.

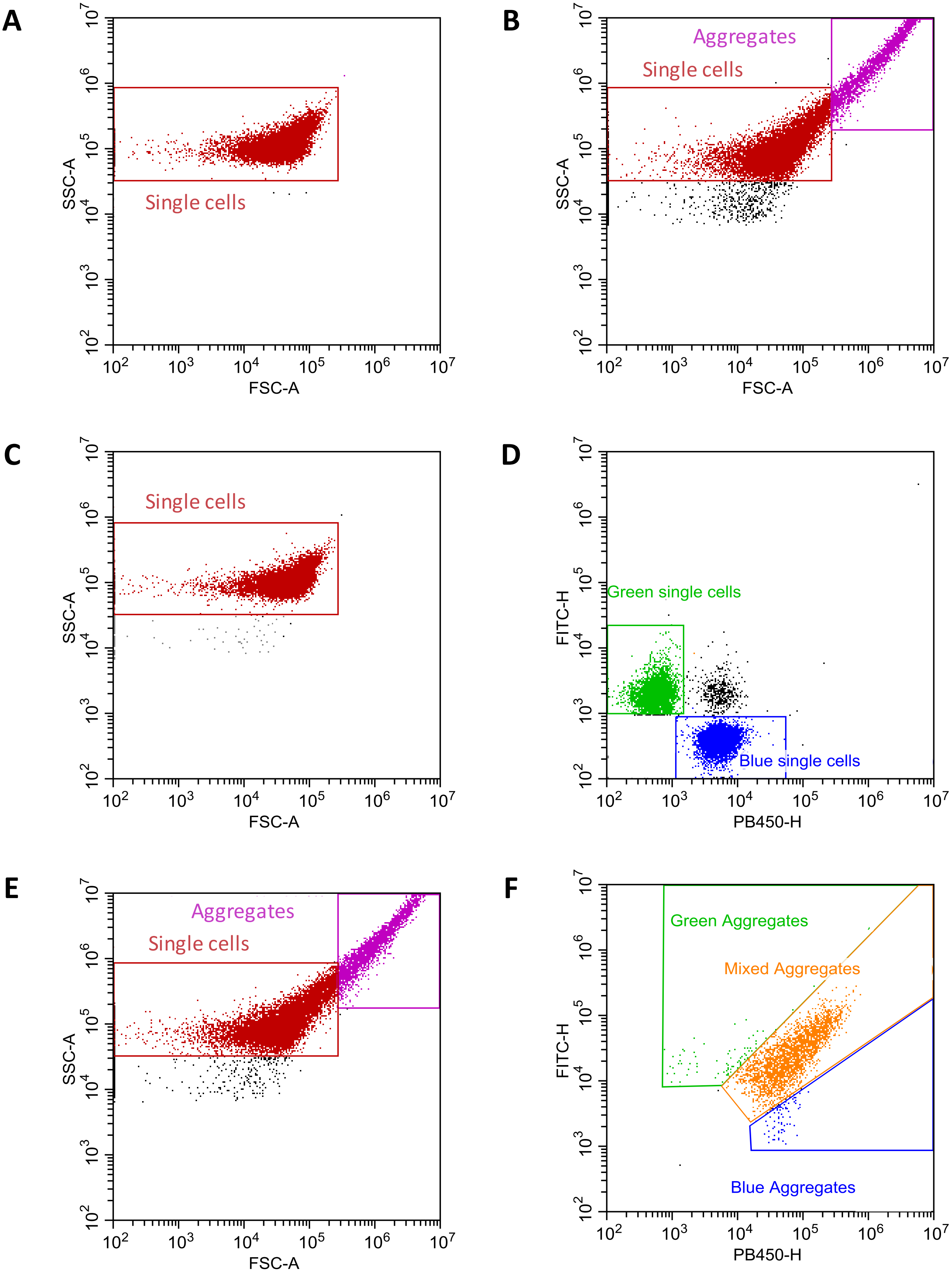
A
Single cells
SSC-A
FSC-A
B
Aggregates
Single cells
SSC-A
FSC-A
C
Single cells
SSC-A
FSC-A
D
Green single cells
Blue single cells
FITC-H
PB450-H
E
Aggregates
Single cells
SSC-A
FSC-A
F
Green Aggregates
Mixed Aggregates
Blue Aggregates
FITC-H
PB450-H

Figure S3

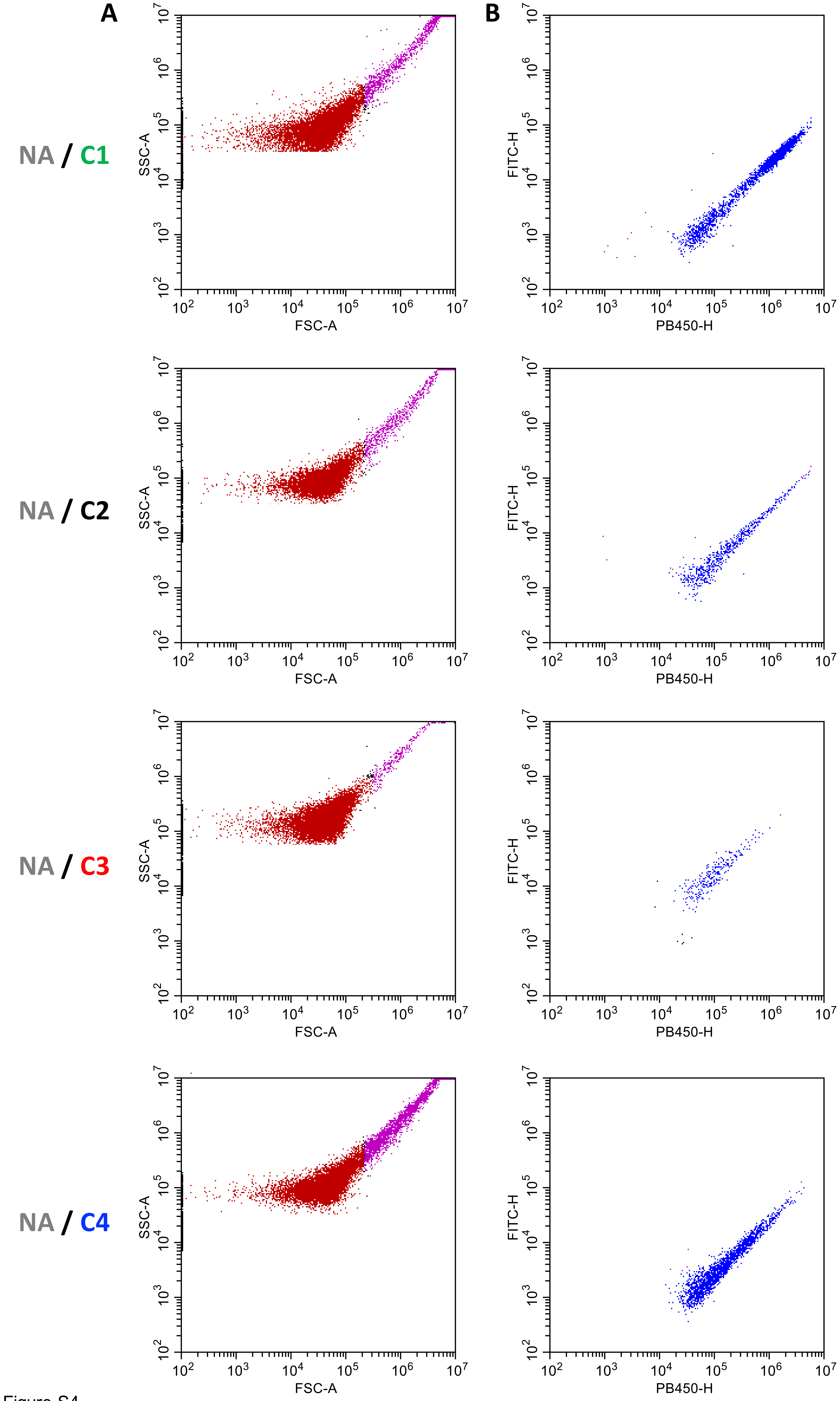
A
B
NA / C1
NA / C2
NA / C3
NA / C4
SSC-A
FSC-A
FITC-H
PB450-H
10^2
10^3
10^4
10^5
10^6
10^7

Figure S4

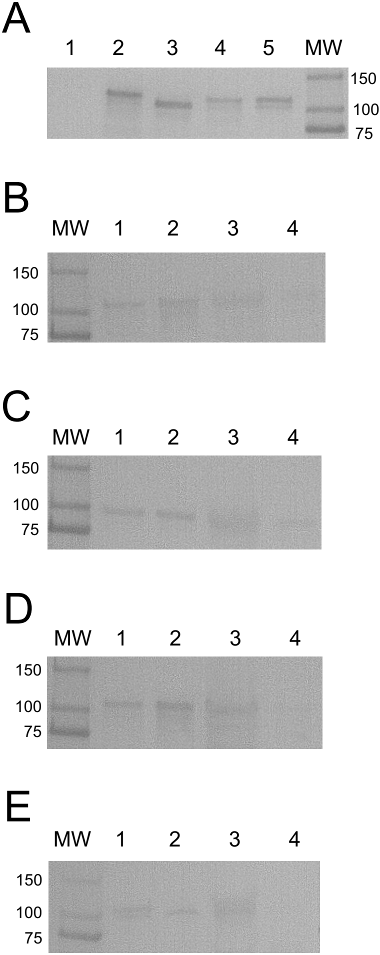
A
1
2
3
4
5
MW
150
100
75
B
MW
1
2
3
4
150
100
75
C
MW
1
2
3
4
150
100
75
D
MW
1
2
3
4
150
100
75
E
MW
1
2
3
4
150
100
75

Figure S5

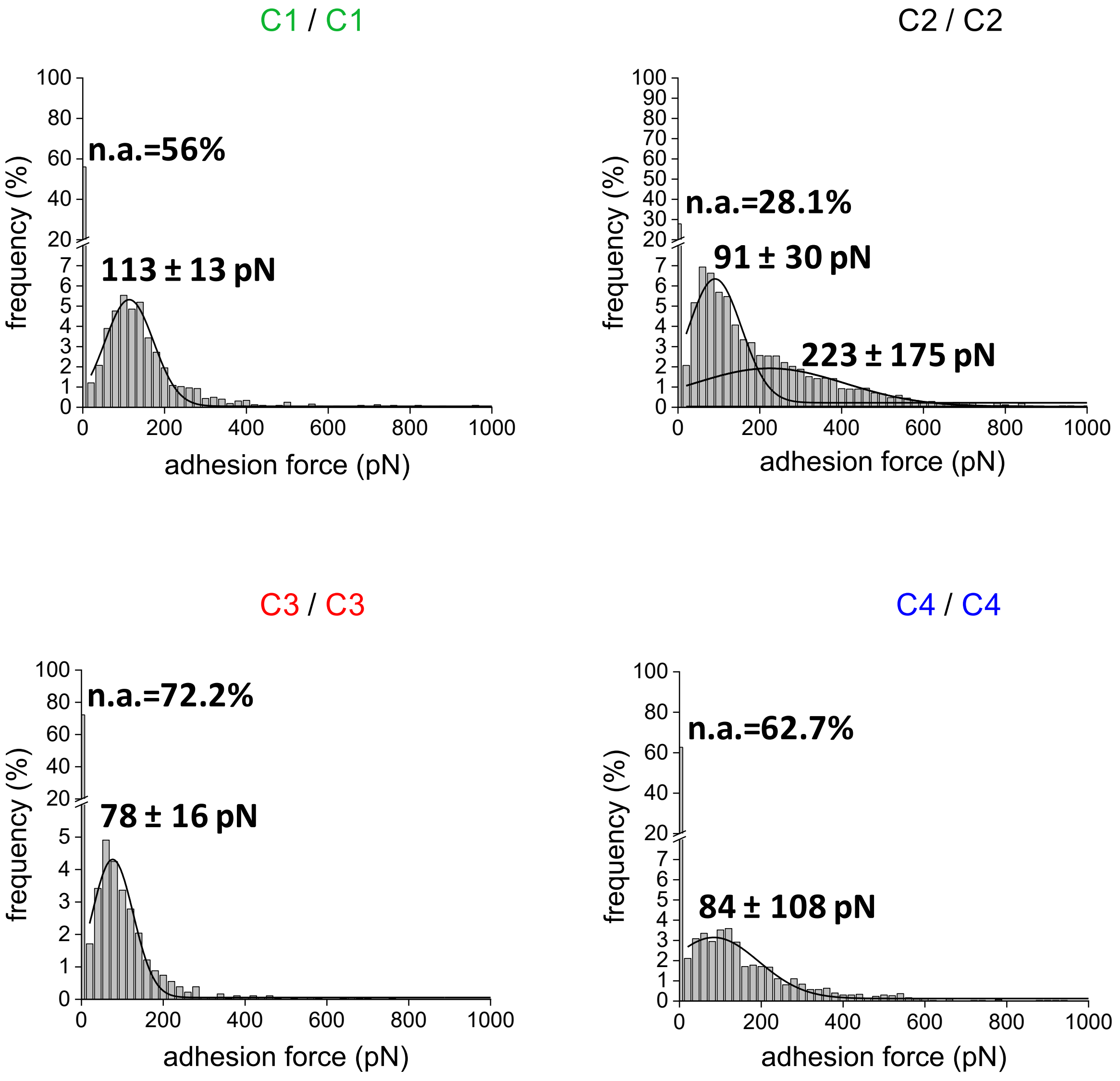
C1 / C1
n.a.=56%
113 ± 13 pN
frequency (%)
adhesion force (pN)
C2 / C2
n.a.=28.1%
91 ± 30 pN
223 ± 175 pN
frequency (%)
adhesion force (pN)
C3 / C3
n.a.=72.2%
78 ± 16 pN
frequency (%)
adhesion force (pN)
C4 / C4
n.a.=62.7%
84 ± 108 pN
frequency (%)
adhesion force (pN)

Figure S6

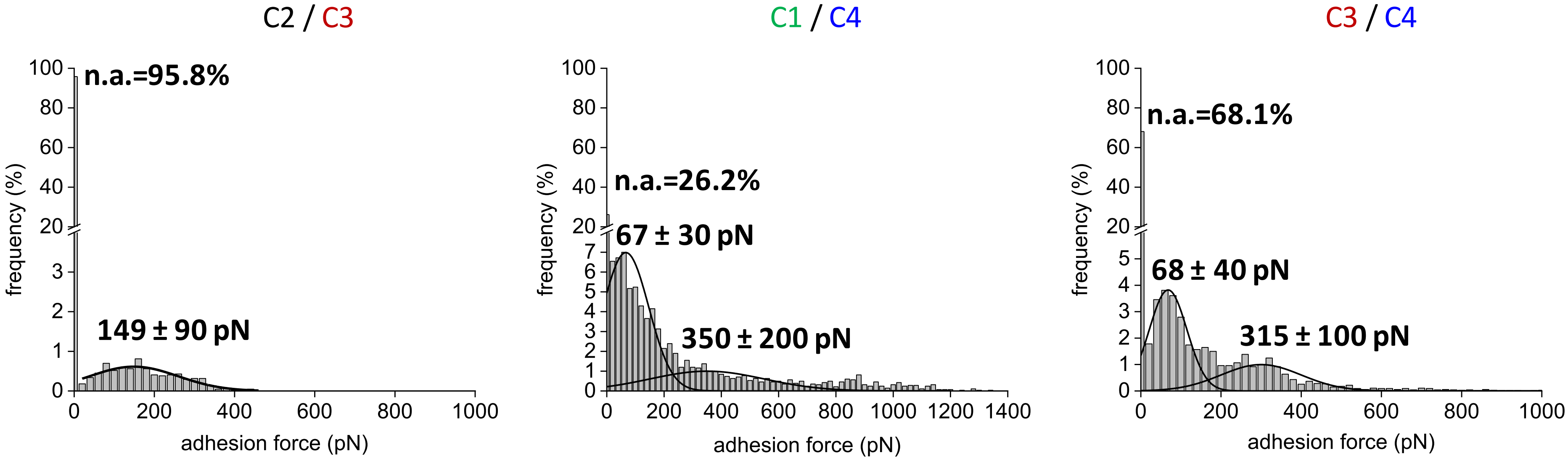

C2 / C3
C1 / C4
C3 / C4
n.a.=95.8%
149 ± 90 pN
n.a.=26.2%
67 ± 30 pN
350 ± 200 pN
n.a.=68.1%
68 ± 40 pN
315 ± 100 pN
frequency (%)
adhesion force (pN)

Figure S7
